# OSR1 downregulation indicates an unfavorable prognosis and activates the NF-κB pathway in ovarian cancer

**DOI:** 10.1007/s12672-023-00778-0

**Published:** 2023-08-29

**Authors:** Zhong Yu, Ling Ouyang

**Affiliations:** Department of Obstetrics and Gynecology, Shengjing Hospital of China Medical University, No. 36, Sanhao Street, Heping District, Shenyang, 110004 Liaoning China

**Keywords:** OSR1, Downregulation, Unfavorable prognosis, NF-κB pathway, Ovarian cancer

## Abstract

**Background:**

Odd-skipped related 1 (OSR1) has been reported as a tumor suppressor gene in various malignant tumors. The mechanism through which OSR1 regulates ovarian cancer (OC) progression remains unclear.

**Materials and methods:**

Immunohistochemistry was utilized to evaluate OSR1 expression in patients with ovarian cancer. We investigated the association between clinicopathological parameters and OSR1 expression in OC patients and the influence of OSR1 expression on patient survival and prognosis. OC cells with OSR1 overexpression or knockdown were established and validated using Western blot and Quantitative reverse-transcription polymerase chain reaction (qRT-PCR). The influence of OSR1 on the NF-κB pathway was examined by analyzing the p-IκBα, IκBα, p65, and p-p65 protein expression. In vitro assays, such as cell cycle assay, Cell Counting Kit-8 (CCK-8), transwell invasion assay, wound healing migration assay, enzyme-linked immunoassay (ELISA), and Annexin V/PI flow cytometry apoptosis assay, were conducted to explore the effect of OSR1 knockdown or dual inhibition of OSR1 and the NF-κB pathway on OC malignant biological behavior.

**Results:**

OSR1 expression was downregulated in OC tissues, with significant associations observed between its expression and The International Federation of Gynecology and Obstetrics (FIGO) stage and tissue differentiation. Low OSR1 expression in OC patients correlated with reduced overall survival (OS) rates and poor prognosis*. *In vitro*,* experiments confirmed a negative correlation between OSR1 expression and NF-κB pathway activity. OSR1 knockdown facilitated OC cell malignant biological behavior, while the NF-κB pathway inhibitor (Bay 11-0782) reversed the impacts of OSR1 knockdown on cell proliferation, migration, invasion, and apoptosis.

**Conclusion:**

Our findings indicate that OSR1 is downregulated and associated with OC prognosis. OSR1 suppresses NF-κB pathway activity and inhibits OC progression by targeting the NF-κB pathway.

**Supplementary Information:**

The online version contains supplementary material available at 10.1007/s12672-023-00778-0.

## Introduction

Ovarian cancer (OC) is the most deadly gynecological malignancy with the highest mortality rate [1]. Most women are diagnosed at an advanced stage due to nonspecific symptoms, resulting in a 30–40% global 5-year survival rate [2, 3]. The onset and progression of OC involve a complex process, and its underlying pathogenetic mechanisms are mainly unknown.

OSR1 is a zinc finger transcription factor encoded by the OSR1 gene located on human chromosome 2p24.1 [4]. Research has revealed that OSR1 is crucial in mesoderm development. OSR1 can regulate embryonic development through multiple mechanisms essential for organ development, such as a limb, kidney, heart, and tongue [5–9]. Recently, OSR1 has been reported to regulate the onset and development of various cancers. Current studies have confirmed that OSR1 is significantly downregulated in cancers, including lung, breast, colon, gastric, kidney, and tongue squamous cell carcinoma (TSCC), and is a tumor suppressor [10–15].

Previous studies have shown that OSR1 inhibits cancer development and progression through several signaling pathways. OSR1 can stimulate the P53 pathway and disrupt the Wnt/β-catenin pathway, which inhibits the growth of gastric cancer, arrests the cell cycle, and triggers apoptosis [13]. Additionally, OSR1 suppresses the Wnt/β-catenin pathway by upregulating GSK3 expression and inhibiting SOX9 expression, which reduces lung cancer invasion and proliferation [16]. OSR1 downregulates Wnt signaling pathway activation in breast cancer [11]. In addition, by blocking the PI3K/Akt and MAPK pathways, OSR1 inhibits colon cancer cell proliferation, invasion, and migration [12]. In TSCC, OSR1 inhibited NF-κB pathway activity [15]. The NF-κB signaling pathway is an important biological signaling pathway implicated in the development of ovarian cancer [17, 18]. NF-κB pathway constitutive activation is associated with poor differentiation, tumor invasion, chemoresistance, late FIGO stage, and poor OS in OC [19, 20].The downstream signaling pathways involved in the regulation of ovarian cancer pathogenesis by OSR1 have not been reported yet.

In the current study, we first used clinical data to identify the relationship between OSR1 expression and clinical pathological characteristics of individuals with OC. This study also intends to investigate the association between OSR1 expression and the prognosis for OC. Moreover, previous research has not examined the relationship between OSR1 and the NF-κB pathway in OC. We sought to understand further how the NF-κB pathway influences the proliferation, apoptosis, invasion, and migration of OC by OSR1.

## Materials and methods

### Specimen source and clinical data

Eighty-six malignant ovarian epithelial tumor samples and 40 normal ovarian tissue samples were analyzed for immunohistochemistry. These samples were obtained from the archived paraffin blocks of surgical specimens from inpatients at Shengjing Hospital of China Medical University between 2014 and 2016. Informed consent was received, and all procedures adhered to the guidelines and regulations set forth by the Ethics Committee of Shengjing Hospital of China Medical University (2021PS823K).Normal ovarian tissue samples were sourced from patients undergoing hysterectomy and preventive oophorectomy due to benign cervical or uterine tumors. They were pathologically confirmed to be normal ovarian tissues without lesions. The 86 OC patients had not received chemotherapy, radiotherapy, or hormone therapy before surgery, and their records were complete, including age, histological type, FIGO stage, histological differentiation grade, lymph node metastasis, survival time, and survival status. Regarding clinical application, surgical pathological staging adheres to the guidelines established by the FIGO in 2009. Patients were followed up until September 1, 2021. OS was the time from the initial diagnosis to death or the last follow-up (censored data for live patients).

### Cell culture and transfection

In this study, normal human ovarian surface epithelial cells (HOSEpiCs) were grown at 37 °C in an ovarian surface epithelial cell medium. Ovarian cancer cells, including A2780, SKOV3, OVCAR3, and COC1, were procured from iCell Bioscience (China). COC1, A2780, and OVCAR3 were cultured in RPMI-1640 medium (Solarbio, China), while SKOV3 cells were cultured in McCoy's 5A medium (Procell, China) with 10% FBS at 37 °C in 5% CO_2_. SKOV3 and OVCAR3 cells were selected for overexpression transfection. To overexpress OSR1, the pcDNA3.1( +) vector (GenScript Biotech, China) was made by inserting the coding sequences of OSR1. The pcDNA3.1( +) empty vector was used as a negative control. G418 (400 µg/mL) was added to screen cell lines with stable transfection.

For OSR1 knockdown, the OSR1 interfering sequences (siRNA1: 5’-GUGUCAAGAGUGUGGGAAATT-3'; siRNA2: 5’-AGAAGGAAUUCGUCUGCAATT-3'; siRNA3: 5’-CCAGAAAAGAAGCCCACAATT-3') and negative control s-iNC:(5’-UUCUCCGAACGUGUCACGUTT-3') were designed and obtained from General bio (China). Cells were transfected with OSR1 overexpression plasmid, the empty vector, OSR1-siRNA, and si-NC using Lipofectamine 3000 (Invitrogen, USA). The Bay 11-7082 was purchased from Aladdin Industrial Corporation (China) and used in the investigation at a concentration of 20 µM [21].

### Immunohistochemistry

After routine deparaffinizing, rehydrating, hydrogen peroxide blocking, and retrieving tissue antigen with a microwave, the sections were incubated with rabbit polyclonal antibody to OSR1 (A18272, 1:50; Abclonal, China) at 4 °C overnight. They were stained with goat anti-rabbit IgG (H + L) HRP (31460, 1:500; Thermo Fisher Scientific, US). Diaminobenzidine solution (DA1010, Solarbio, China) was employed to counterstain, and the sections were subsequently treated with hematoxylin (H8070, Solarbio) for 1 min and dehydrated. Two pathologists, unaware of the clinical context, evaluated the immunostained tissue sections. One hundred cells were counted in each of the five view fields on each slide at 400 × magnification. The intensity of IHC staining was visualized and scored as 3 (strong stain), 2 (medium), 1 (weak), and 0 (no stain). The extent of staining ranged from 0 to 4, corresponding to the immune-reactive tumor cells percentage (76–100%, 51–75%, 26–50%, 1–25%, 0%), with 4 being the highest. Based on the staining intensity and staining scores, each sample was assigned a score between 0 and 12, categorizing it into two categories: OSR1 low expression (0–6) and OSR1 high expression (8–12) [22].

### Quantitative real-time PCR

Total cell RNA was obtained using TRIpure lysate (BioTeke, China). Quantification and reverse transcription of RNA was carried out using the PCR system (Bioneer, Korea). The 2 ^–ΔΔCT^ method was employed for quantifying gene expression, with β-actin as the internal control. Primer sequences utilized in this study comprised OSR1-F: CTCCTTCCTTCAGGCAGTG; OSR1-R: ATCTCGGGCTTGGGTTG; β-actin-F: CAGCAAGCAGGAGTATGACG; and β-actin-R: TTAGGATGGCAAGGGACTTC.

### western blot (wb)

OC cells lysis with PMSF containing lysis buffer (Beyotime, China) and then centrifugation at 10,000 g for 5 min at 4 °C was done to collect the supernatant. A BCA Protein Assay Kit (Beyotime) was employed for protein quantification. The protein separation was performed via SDS-PAGE (Beyotime, China), followed by PVDF membranes (Millipore, MA, USA) transfer and blocking for 1 h with non-fat milk. Primary antibodies were incubated with membranes at 4 °C overnight. After TBST washing, secondary antibodies (1:5000) were incubated with membranes for 45 min at 37 °C, then washed with TBST. ECL reagents (Beyotime, China) were used for blot visualization. Primary antibodies included OSR1, PCNA, and cyclin D1 from ABclonal, China; caspase-3, cleaved-caspase-3, Bcl-2, and Bax from CST, USA; p-p65, p65, p-IκBα, and IκBα from Wanleibio, China. Secondary antibodies used were goat anti-rabbit IgG and goat anti-mouse IgG from Beyotime.

### CCK-8 cell viability assay

OSR1-overexpressing SKOV3 and OVCAR3 cells (3 × 10^3^) in 96-well plates were grown for 12, 24, 36, and 48 h. Each well was then supplemented with 10 µL of CCK-8 reagent (Beyotime). Cells were then incubated for 2 h. Cell viability was calculated by the optical density at 450 nm in a microplate reader.

### Cell cycle assay

Cell Cycle Detection Kits (Beyotime) were used to assess cell cycle progression. 5 × 10^5^ cells were grown in 6-well plates and fixed for 12 h at 4 °C in 70% cold ethanol, followed by staining with PI staining solution containing RNase A in the dark for 30 min. Cells were counted using a flow cytometer.

### Migration assay

A wound-healing test was conducted to assess OC cells' migration capacity. The media was replaced with 20 µg/mL of mitomycin C (Sigma, MO, USA) containing serum-free medium (SFM). A 200-µL pipette tip was used to create the wounds. The cells were then cultured for 48 h after being washed with SFM. The migration rate was calculated using images taken at 0 and 48 h.

### Transwell invasion assay

The invasive potential of transfected OC cells was assessed using a Matrigel-coated Transwell chamber (Corning, NY, USA). The cells were added to the upper compartment, while the lower section was filled with the 10% FBS-containing cell culture medium. Invaded cells were counted following the fixation and staining with 0.5% crystal violet (Amresco, USA) after 48 h. Under an inverted microscope (200x), cells were counted as they invaded the lower layer. For each sample, 5 fields were chosen to quantify the number of cells, and the average value was calculated.

### ELISA assay

MMP-9 and MMP-2 expression levels were measured using the human MMP-2 and MMP-9 ELISA kit (MultiSciences, China) following the manufacturer's instructions.

### Flow cytometry for apoptosis detection

The Annexin V-FITC Apoptosis Detection Kit (KeyGEN, Nanjing, China) was employed to examine cell apoptosis. A binding buffer containing AnnexinV-FITC was used to resuspend cells (5 × 10^5^) cultured in 6-well plates. The PI staining solution was used to stain the cells for 15 min in the dark. The flow cytometer was used to analyze the levels of apoptosis.

### Hoechst staining assay

After fixing for 10 min and PBS washing, the transfected cells were stained with Hoechst staining solution (Beyotime) for 5 min. Cells were mounted after being washed in PBS, then images were captured using a Fluorescent microscope (IX53, OLYMPUS, Japan).

### Statistical analysis

Statistical analysis was conducted using SPSS 26.0 and GraphPad Prism version 9.0. A two-tailed student's t-test was employed to compare two groups, and a one-way single factor analysis of variance (ANOVA) for more than two groups. The chi-square test was used to analyze the differences between counting data groups. Survival curves were constructed using the Kaplan–Meier (KM) method in SPSS, with the log-rank test comparing curve differences. Through univariate and multivariate analysis, the Cox regression analysis was applied to examine the effect of clinicopathological factors on the prognosis for OC. A p-value below 0.05 indicated statistical significance (P < 0.05, *; P < 0.01, **; P < 0.001, ***; P < 0.0001, ****).

## Results

### Expression and clinical significance of OSR1 in ovarian tissues

**Fig. 1:**
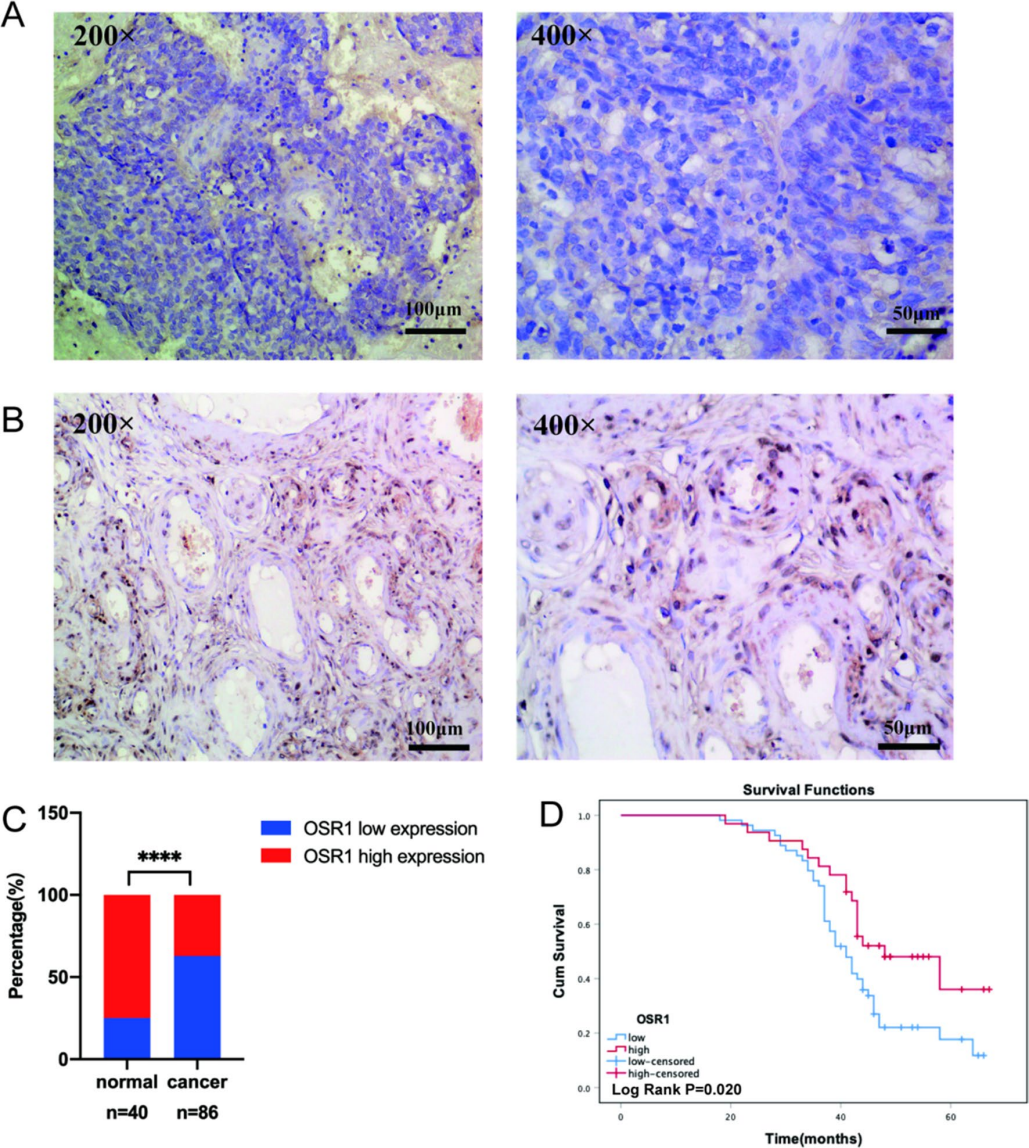
Low expression of OSR1 was shown in ovarian cancer tissues and was correlated with worse OS. **A** The representive image of OSR1 low expression in OC tissue by IHC (200 × and 400 ×); **B** The representive image of OSR1 high expression in normal ovarian tissue by IHC (200 × and 400 ×); **C** Statistical diagram of OSR1 expression rate in ovarian cancer group and normal ovarian group(****P < 0.0001);** D** KM survival curve analysis for OSR1 expression in 86 patients with OC(Log Rank P = 0.02)

Immunohistochemical staining of paraffin-embedded sections was conducted to assess the OSR1 expression in patients with ovarian cancer. Our IHC data demonstrated a significant reduction of OSR1 expression in OC tissues to normal ovarian tissues (Fig. 1A, B). OSR1 was primarily localized in the cytoplasm and partially localized in the nucleus. Of 86 OC patients, 32 cases (37.2%) showed high OSR1 expression, while 54 cases (62.8%) displayed low OSR1 expression. In the 40 normal ovarian tissue samples, the increased expression rate of OSR1 was 75% (30/40), and the low expression rate was 25% (10/40). The low OSR1 expression rate in the ovarian cancer group was notably higher than the normal ovarian group (P < 0.0001, Fig. 1C).

86 OC cases were monitored until September 2021 for survival analysis. Fifty-nine patients succumbed to the disease during the follow-up period, with a median OS of 43.0 months. Patients exhibiting low and high OSR1 expression had median survival times of 41 and 48 months, respectively. KM survival curves indicated that patients showing low OSR1 expression had notably shorter OS than those with high OSR1 expression, suggesting an association between low OSR1 expression in OC patients and poor prognosis (Log Rank P = 0.020, Fig. 1D).

As shown in Table 1, the association between OSR1 expression and patients' clinicopathological features was assessed. OSR1 expression in OC was negatively linked with the FIGO stage (P = 0.025) and histological differentiation (P = 0.038). OSR1 expression and other clinicopathological characteristics were not found to be significantly correlated. Through Cox regression analysis, the relative risk factors affecting the prognosis of OC patients were further investigated. As presented in Table 2, low OSR1 expression (P = 0.025), FIGO stage (P < 0.001), histological differentiation (P = 0.021), and lymph node metastasis (P < 0.001) may affect the OS of OC patients based on Univariate analysis results. Multivariate analysis revealed that the FIGO stage (P < 0.001) and Lymph node metastasis (P < 0.001) could serve as independent prognostic factors; however, OSR1 expression was not found to be significant (P = 0.194). According to our findings, OSR1 levels were lower in OC tissues, which was associated with a poor prognosis for OC.

**Table 1:** Association between OSR1 expression and clinicopathologic characteristics of OC patients

Characteristic	Cases (%)	OSR1 expression		χ2	P
		Low	High		
Age					
< 55	34 (39.5)	18	16	2.335	0.127
> 55	52 (60.5)	36	16		
FIGO stage					
I-II	23 (26.7)	10	13	5.012	0.025
III-IV	63 (73.3)	44	19		
Differentiation					
Well-Moderate	31 (36.0)	15	16	4.30	0.038
Poor	55 (64.0)	39	16		
Lymphatic metastasis					
No	50 (58.1)	31	19	0.043	0.979
Yes	33 (38.4)	21	12		
Unknown	3 (3.5)	2	1		
Pathological type					
Serous	63 (73.3)	40	23	1.24	0.74
Mucinous	8 (9.3)	6	2		
Endometrioid	10 (11.6)	5	5		
Clear cell carcinoma	5 (5.8)	3	2

**Table 2:** Univariate and multivariate analyses of various prognostic parameters in OC patients

	Univariate analysis			Multivariate analysis		
	Hazard ratio	95% CI	P	Hazard ratio	95% CI	P
Age	1.696 (0.982–2.929)	0.982–2.929	0.058			
Pathological type	1.111 (0.85–1.453)	0.85–1.453	0.44			
FIGO stage	9.182 (3.53–23.885)	3.53–23.885	< 0.001	6.857 (2.504–18.777)	2.504–18.777	< 0.001
Differentiation	1.936 (1.106–3.389)	1.106–3.389	0.021	1.729 (0.952–3.139)	0.952–3.139	0.072
Lymphatic metastasis	3.425 (2.149–5.457)	2.149–5.457	< 0.001	2.501 (1.451–4.312)	1.451–4.312	< 0.001
OSR1	0.523 (0.297–0.922)	0.297–0.922	0.025	0.68 (0.379–1.217)	0.379–1.217	0.194

### OSR1 negatively regulates the NF-κB pathway in OC cells

The NF-κB pathway in ovarian cancer is found to be constitutively activated [23]. The expression of NF-κB pathway protein p-p65 and the expression of downstream target genes controlled by NF-κB pathway are increased, promoting the malignant aggressiveness of ovarian cancer [24]. In inactive condition, p65 is tightly bound to its inhibitor protein IκBα and isolated in the cytoplasm [25]. Different forms of stimulation lead to the ubiquitination and degradation of IκBα, which phosphorylates p65 and causes it to be transported from the cytoplasm to the nucleus, where it interacts with the promoter of its target genes [26].

**Fig. 2:**
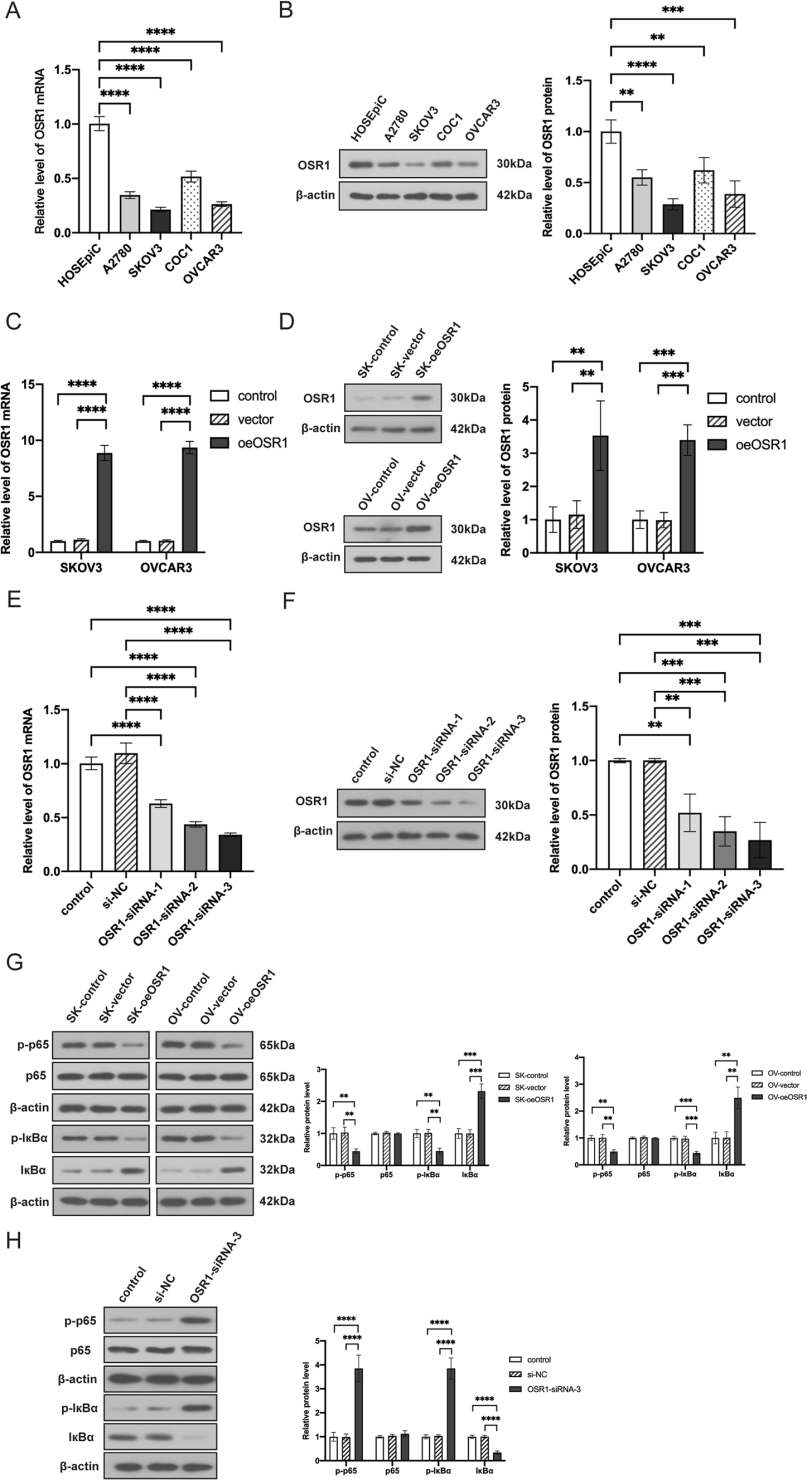
OSR1 inhibited the NF-κB pathway. **A–B** Protein and mRNA expression levels of OSR1 in HOSEpiC and OC cells, including SKOV3, OVCAR3, COC1, and A2780; **C–D** Results of OSR1 protein and mRNA expression levels in SKOV3 and OVCAR3 cells with overexpressed OSR1; **E–F** Results of OSR1 protein and mRNA levels in A2780 cells transfected with OSR1-siRNA; **G** WB determined the p-IκBα, IκBα, p65, and p-p65 protein expression in OSR1 overexpressed SKOV3 and OVCAR3 cells; **H** WB determined the p-IκBα, IκBα, p65, and p-p65 protein expression in OSR1 knockdown A2780 cells. *P < 0.05; **P < 0.01;***P < 0.001;****P < 0.0001. Data shown represent the mean ± SD from three independent experiments rom three independent experiments, each performed in triplicate

As depicted in Fig. 2A, B, compared to the ovarian epithelial cell line HOSEpiC, markedly reduced protein and mRNA levels of OSR1 were seen in all OC cell lines. Furthermore, OSR1 expression in OVCAR3 and SKOV3 cells was lesser than in COC1 and A2780 cells. We then transfected the OSR1 overexpression plasmid into OVCAR3 and SKOV3 cells and transfected OSR1-siRNA into A2780 cells. Protein and mRNA levels of OSR1 were substantially increased in OSR1-overexpressed SKOV3 and OVCAR3 cells (Fig. 2C, D). OSR1 protein and mRNA levels were notably reduced in OSR1-knockdown A2780 cells (Fig. 2E, F). WB results demonstrated that in OSR1-overexpressed SKOV3 and OVCAR3 cells, the p-p65 and p-IκBα expression was substantially downregulated, the expression of total p65 remained unchanged, and the expression of IκBα was notably elevated (Fig. 2G). The expression of p-p65 and p-IκBα increased significantly in A2780 cells transfected with OSR1-siRNA-3, whereas total p65 expression remained unchanged, and IκBα expression was substantially downregulated(Fig. 2H). These findings indicated that OSR1 suppressed the activity of the NF-κB pathway.

### OSR1 regulates OC proliferation and cell cycle through the NF-κB pathway

**Fig. 3:**
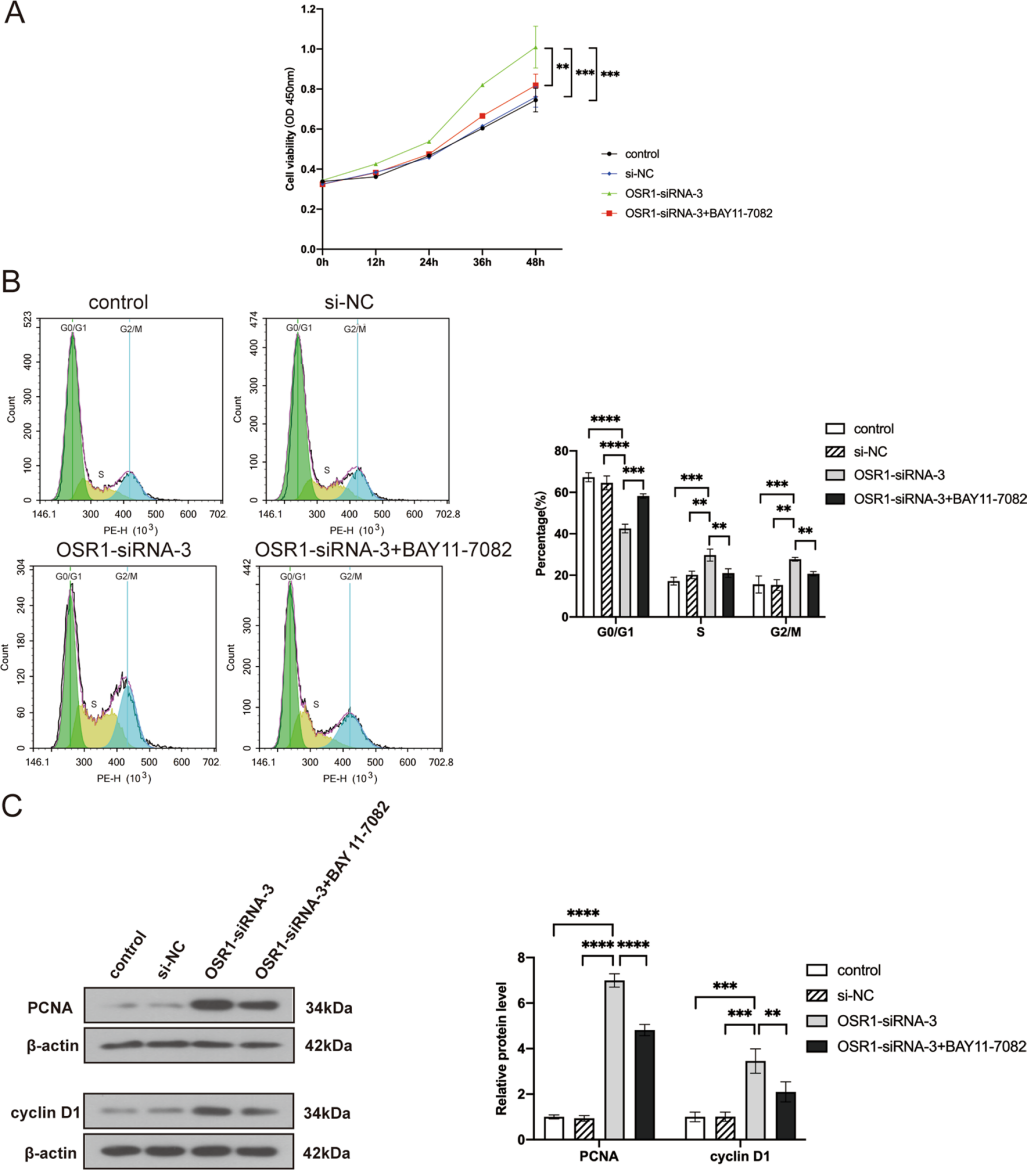
The effects of double inhibition of OSR1 and NF-κB pathway on the proliferation and the cell cycle of OC cells. **A** CCK-8 assay demonstrated that OSR1 knockdown promoted OC cell growth activity, which could be reversed by BAY 11-7082. **B** Flow cytometry assay suggested OSR1 knockdown promoted OC cell cycle progression, which could be reversed by BAY 11-7082. **C** WB determined the expression of PCNA and cyclinD1 after OSR1 knockdown or double inhibition of OSR1 and NF-κB pathway. **P < 0.01;***P < 0.001;****P < 0.0001. Data shown represent the mean ± SD from three independent experiments rom three independent experiments, each performed in triplicate

To further investigate whether OSR1 inhibits OC malignant biological behavior by suppressing the NF-κB pathway, OSR1, and the NF-κB pathway were simultaneously inhibited in A2780 cells. BAY 11-7082 treatment was used to inhibit the NF-κB pathway. BAY 11-7082 is a common inhibitor of the NF-κB pathway [27]. Moreover, it has been shown to reduce the proliferation, migration, and invasion of OC cells and promote apoptosis [28]. CCK-8 assay demonstrated that OSR1 knockdown substantially promoted the growth of A2780 cells at 48 h compared with the control and si-NC groups. However, when we doubly inhibited OSR1 and the NF-κB pathway, cell proliferation activity at 48 h was significantly decreased compared with OSR1 knockdown alone (Fig. 3A). Flow cytometry assay results demonstrated that the proportion of cells in the G0/G1 phase declined while the proportion of cells in the S and G2/M phases rose in OC cells transfected with OSR1-siRNA-3 alone, but BAY 11-7082 treatment could partially reverse those changes (Fig. 3B). WB results showed increased expression of cyclin D1 protein and proliferating cell nuclear antigen (PCNA) following OSR1 knockdown. Compared with the OSR1-siRNA-3 group, cyclin D1 and PCNA expression in the OSR1-siRNA-3 + BAY 11-7082 group was significantly decreased, confirming that OSR1 regulated OC proliferation by mediating the NF-κB pathway (Fig. 3C).

### OSR1 regulates OC invasion and migration through the NF-κB pathway

**Fig. 4:**
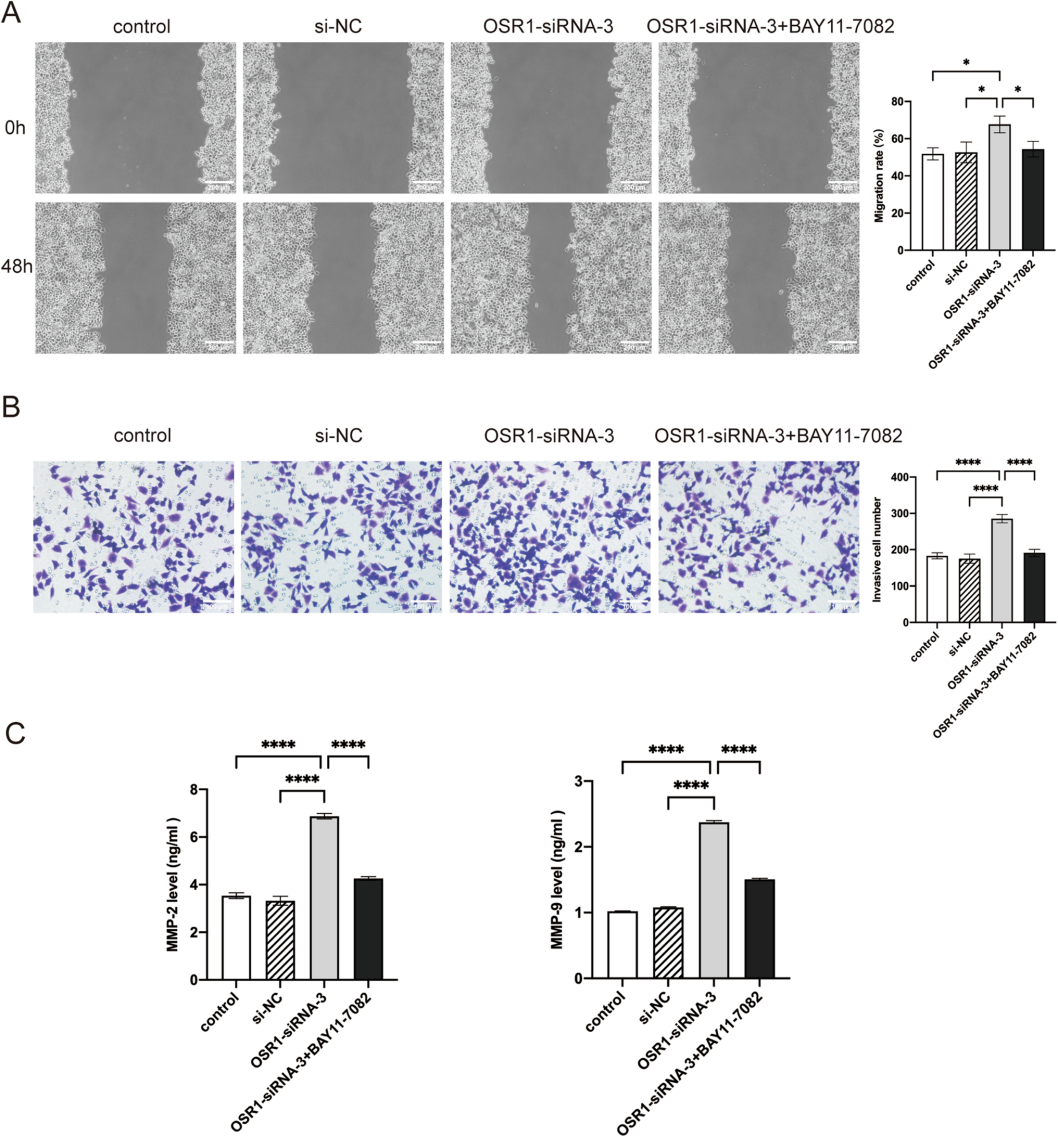
The effects of double inhibition of OSR1 and NF-κB pathway on the invasion and migration of OC cells. **A** The wound healing assay demonstrated that OSR1 knockdown promoted OC migration in A2780 cells, which was reversed by BAY 11-7082 (× 100). **B** Transwell assay demonstrated that OSR1 knockdown suppressed invasion of OC cells, which was reversed by BAY 11-7082 (× 200). **C** ELISA assay detected the MMP-9 and MMP-2 expression in the four groups. *P < 0.05; ****P < 0.0001. Data shown represent the mean ± SD from three independent experiments rom three independent experiments, each performed in triplicate

The effect of OSR1 on OC cell invasion and migration was examined using Transwell invasion and wound healing assays. The migration analysis revealed that A2780 cells transfected with OSR1-siRNA-3 exhibited a more rapid wound healing rate than the control and si-NC groups. Nevertheless, upon simultaneous inhibition of OSR1 and the NF-κB pathway, the migration rate of OC cells decelerated relative to the OSR1-siRNA-3 group (Fig. 4A). The Transwell invasion assay outcomes demonstrated a substantial enhancement in cell invasion capacity following OSR1 knockdown, which was notably mitigated by BAY 11-7082 (Fig. 4B). Moreover when OSR1 was downregulated, ELISA assay results indicated an increase in the expression levels of invasion-linked proteins MMP-9 and MMP-2; however, BAY 11-7082 partially counteracted these alterations (Fig. 4C). The findings suggest that OSR1 inhibits OC cell invasion and migration via the NF-κB pathway.

### Downregulation of OSR1 inhibited OC cell apoptosis via the NF-κB pathway

**Fig. 5:**
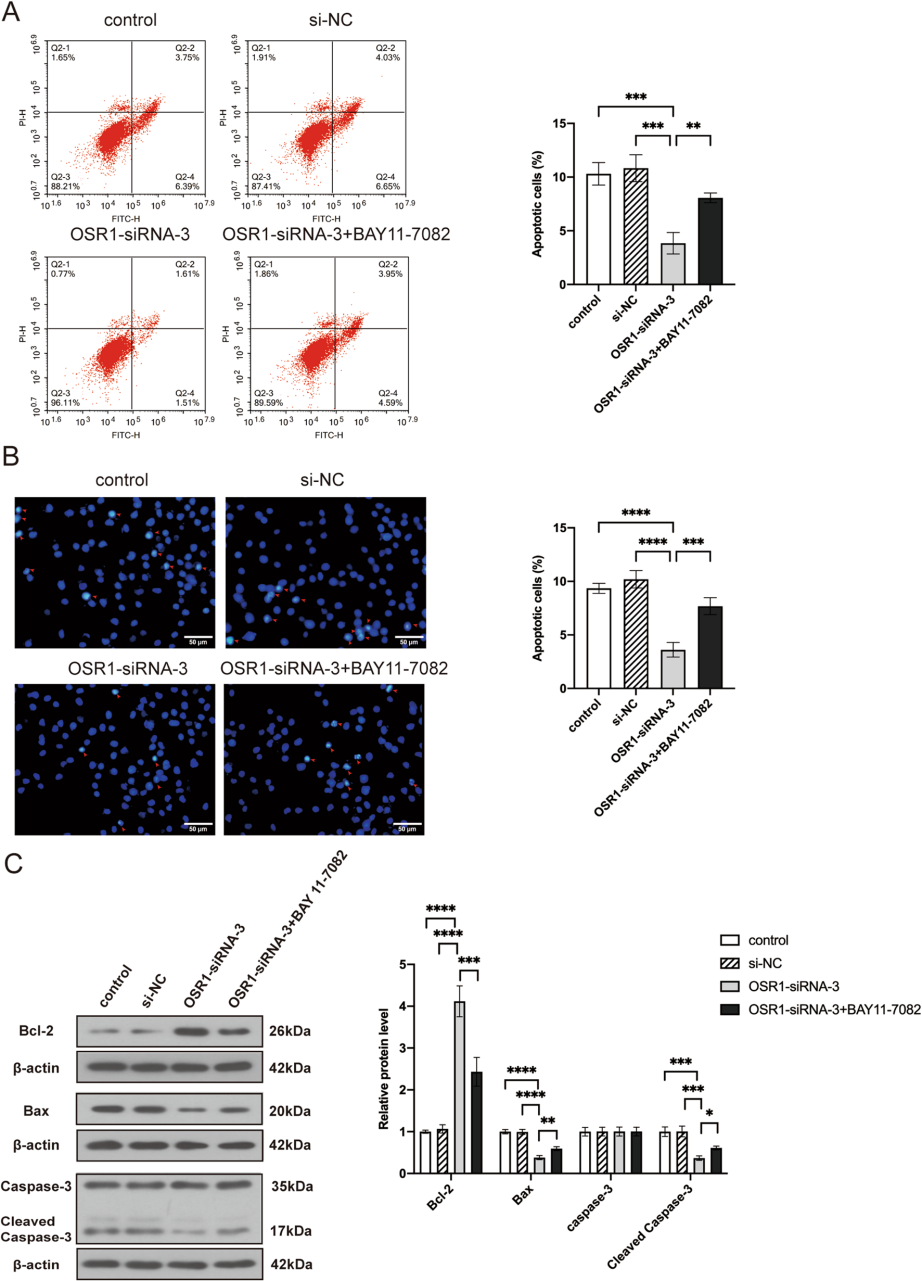
The influences of double inhibition of OSR1 and NF-κB pathway on the apoptosis of OC cells. **A** Annexin V-FITC/PI flow cytometry assay demonstrated that OSR1 knockdown inhibited OC apoptosis, which was reversed by BAY 11-7082. **B** The apoptotic cells detected by hoechst staining assay reduced significantly in A2780 OC cells with OSR1 knockdown but increased when treated the cells with BAY 11-7082. **C** WB detected the Bax, Bcl-2, Caspase-3, Cleaved Caspase-3 protein expression in the four groups. *P < 0.05;**, P < 0.01;***P < 0.001;****P < 0.0001. Data shown represent the mean ± SD from three independent experiments rom three independent experiments, each performed in triplicate

The Annexin V-FITC/PI flow cytometry apoptosis assay and Hoechst staining assay revealed that, compared to the control group and si-NC group, OSR1 knockdown led to reduced cell apoptosis, while BAY 11–7082 significantly counteracted the decline in cell apoptosis ability caused by OSR1 knockdown (Fig. 5A, B). WB analysis displayed a notable rise in Bcl-2 expression and a decline in Bax and Cleaved Caspase-3 expression following OSR1 knockdown, with BAY 11-7082 partially reversing these alterations (Fig. 5C). These findings indicate that OSR1 facilitates OC apoptosis via the NF-κB pathway.

## Discussion

Ovarian cancer ranks as the most lethal malignancy among the three primary gynecologic neoplasms of the reproductive system. At the time of diagnosis, 70% of afflicted individuals present with advanced-stage disease, frequently characterized by extensive metastasis within the pelvic and abdominal regions or even at remote sites [29, 30]. After developing treatment resistance, the patient's prognosis becomes worse [31, 32]. Despite breakthroughs in diagnostic and therapeutic approaches, there remains a pressing need for additional research aimed at identifying innovative molecular markers to facilitate early detection and prognostic prediction.

The OSR1 gene functions as a zinc finger transcription factor crucial for embryonic development. Besides its role in development, OSR1 also serves as a vital tumor suppressor. Our prior findings indicated that OSR1 expression was markedly lower in 30 fresh OC samples compared to 20 normal ovarian tissue samples, and its overexpression could impede ovarian cancer cell proliferation while promoting apoptosis [33]. Building on these findings, immunohistochemical staining was used to assess OSR1 expression in samples from 86 epithelial OC cases and 40 normal ovarian tissue cases. The results revealed significantly reduced OSR1 expression in OC tissues compared to normal ovarian tissues. We gathered the clinicopathological features and survival data of the 86 ovarian cancer patients, and the results indicated a negative relationship between OSR1 expression and FIGO stage, histological differentiation. This study also discovered that OC patients with low OSR1 levels had poorer OS than those with high OSR1 levels. Kaplan–Meier survival analysis demonstrated a relationship between OSR1 expression levels and patient prognosis. Cox regression analysis identified OSR1 as one of the risk factors influencing OC patient prognosis, although it was not an independent prognostic factor. Previous research has shown that OSR1 levels were lower in renal cell carcinoma tissues than in normal renal tissues and are negatively associated with histological differentiation grading [14]. Similarly, OSR1 expression is reduced in lung cancer and is linked to lower histological differentiation [16]. OSR1 downregulation in breast cancer negatively correlates with lymph node metastasis and ER expression [15]. In tongue cancer tissue, OSR1 expression is decreased, and it is even lower in metastatic cases compared to non-metastatic ones [15, 34]. The methylation of the OSR1 promoter leads to the downregulation of *OSR1* gene expression in gastric cancer, and OSR1 methylation level is an independent prognostic marker for gastric cancer patients [13]. In colon cancer patients, reduced OSR1 expression is notably linked with decreased OS and distant metastasis-free survival [12]. These findings demonstrated that, OSR1 is negatively correlated with the malignancy of various tumors. While OSR1 could potentially serve as a prognostic indicator for OC, further validation with larger sample sizes is necessary to confirm our findings. According to studies about the origin and molecular pathogenesis of ovarian cancer, ovarian high-grade serous carcinomas, the most common pathological type with poor prognosis, are considered to have originated in fallopian tube epithelial precursor lesions [35, 36]. Therefore, we will collect fallopian tube epithelium tissues and fallopian tube epithelium cell line to verify the expression of OSR1 in the future.

Numerous pieces of research have established a strong link between the NF-κB pathway and tumor formation, growth, invasion, and metastasis. Constitutive activation of the NF-κB pathway is crucial in OC [17]. NF-κB pathway activity is elevated in OC cells, and NF-κB regulates the genes implicated in proliferation, adhesion, invasion, angiogenesis, and the formation of a pro-inflammatory microenvironment in OC cells [37–39]. NF-κB p65 is primarily highly expressed in the nucleus of OC cells, and its expression level correlates with tissue differentiation and FIGO stages [40]. Over 90% of identified OC patients exhibit increased expression of NF-κB subunits p50 and p65 [27, 41]. Targeting NF-κB in tumor cells has emerged as a new direction for ovarian cancer treatment [42]. By transfecting the OSR1 overexpression plasmid or siRNA, we modulated OSR1 expression to examine its impact on the NF-κB pathway. The results revealed that OSR1 overexpression inhibited the p-IκBα and p-p65 expression in the NF-κB pathway. Concurrently, IκBα, an inhibitory protein of NF-κB dimers, was upregulated, and total p65 protein expression remained unchanged with OSR1 overexpression. When OSR1 expression was inhibited by transfecting siRNA, the IκBα, p-IκBα, and p-p65 expressions were reversed, with p65 protein expression remaining unaltered. By blocking the NF-κB pathway, OSR1 overexpression significantly lowered the invasion and migration of TSCC [15]. Our study's findings indicated that OSR1 could inhibit NF-κB pathway activation, which aligns with previous research on OSR1 in TSCC.

Our in vitro experiment results suggested that downregulated OSR1 expression promoted OC proliferation, cell cycle progression, invasion, metastasis, and inhibited apoptosis. When we simultaneously inhibited OSR1 and the NF-κB pathway, the impacts of decreased OSR1 expression on OC cell malignant behaviors were reversed. Thus, OSR1 could suppress OC progression by the NF-κB pathway regulation. Epigenetic regulation controls OSR1 expression in gastric cancer and lung cacinoma, and mechanically OSR1 is downregulated due to promoter CpG methylation [14, 43, 44]. Whether the downregulation of OSR1 expression in ovarian cancer occurs through the same mechanism warrants further investigation.

## Conclusion

In summary, OSR1 is downregulated in OC and may be a potential indicator of tumor malignancy and prognosis and a possible therapeutic target. Our study is the first to investigate the clinical implication of OSR1 in ovarian cancer. Moreover, OSR1 inhibits the malignant growth, invasion and metastasis of OC cells via the NF-κB pathway.

### Supplementary Information

Additional file1 (DOCX 27138 KB)

## Data Availability

All data and material are available from the corresponding author on reasonable request.

## Abbreviations

OC: Ovarian cancer
OSR1: Odd-skipped related 1
qRT-PCR: Real-time PCR
WB: Western blot
CCK-8: Cell Counting Kit-8
FIGO: Federation International of Gynecology and Obstetrics
OS: Overall survival
TSCC: Tongue squamous cell carcinoma
KM: Kaplan–Meier
ANOVA: Analysis of variance
