# Supplementary material for: OSR1 downregulation indicates an unfavorable prognosis and activates the NF-κB pathway in ovarian cancer
Source: Discov Oncol. 2023 Aug 29;14:159. doi: 10.1007/s12672-023-00778-0 (PMC10465422; doi:10.1007/s12672-023-00778-0)

The original images of Western blot are as follows：

Figure 2B

OSR1

From left to right ： HOSEPiC；A2780；COC1；OVCAR3；


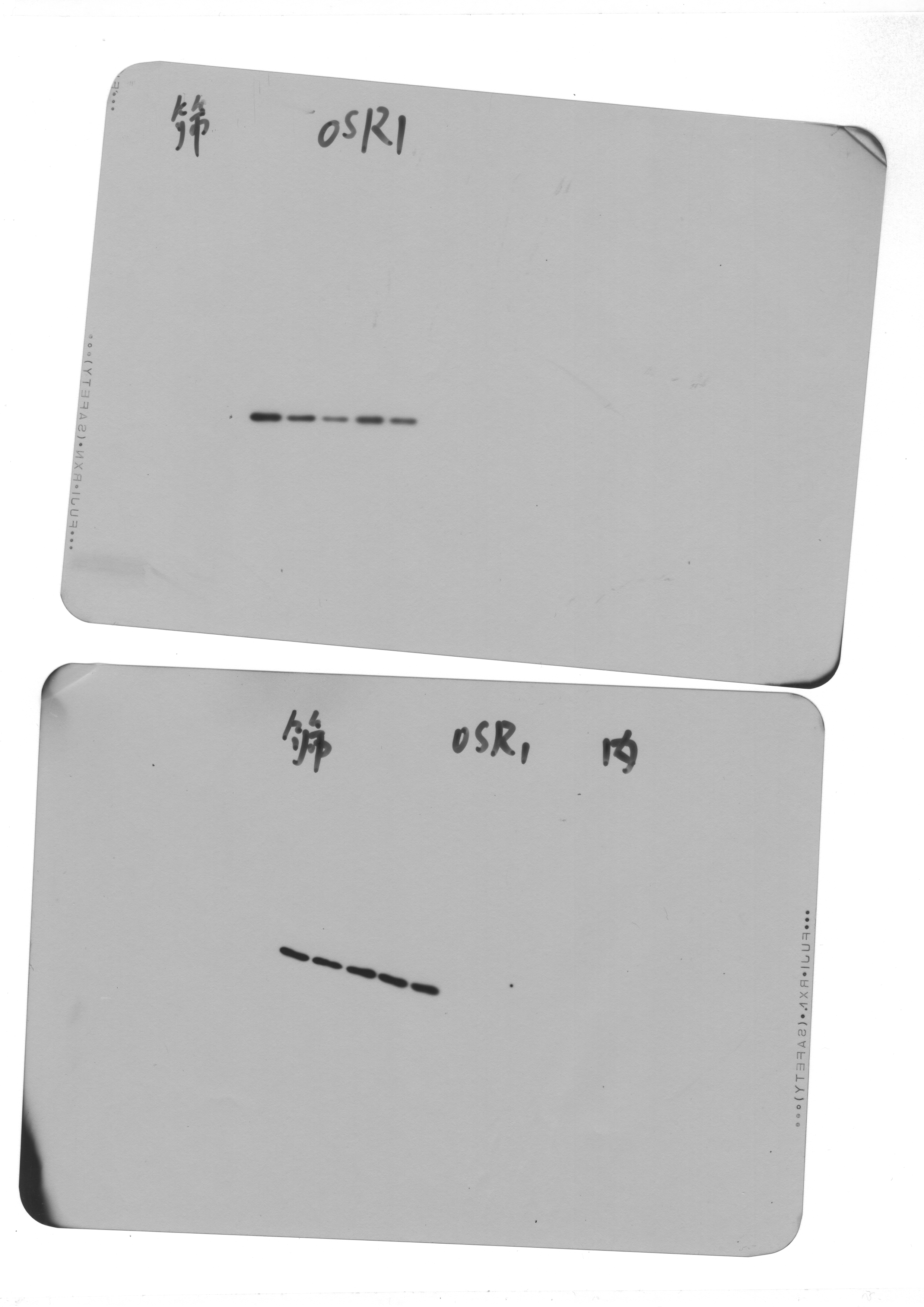


β-actin

From left to right ： HOSEPiC；A2780；COC1；OVCAR3；


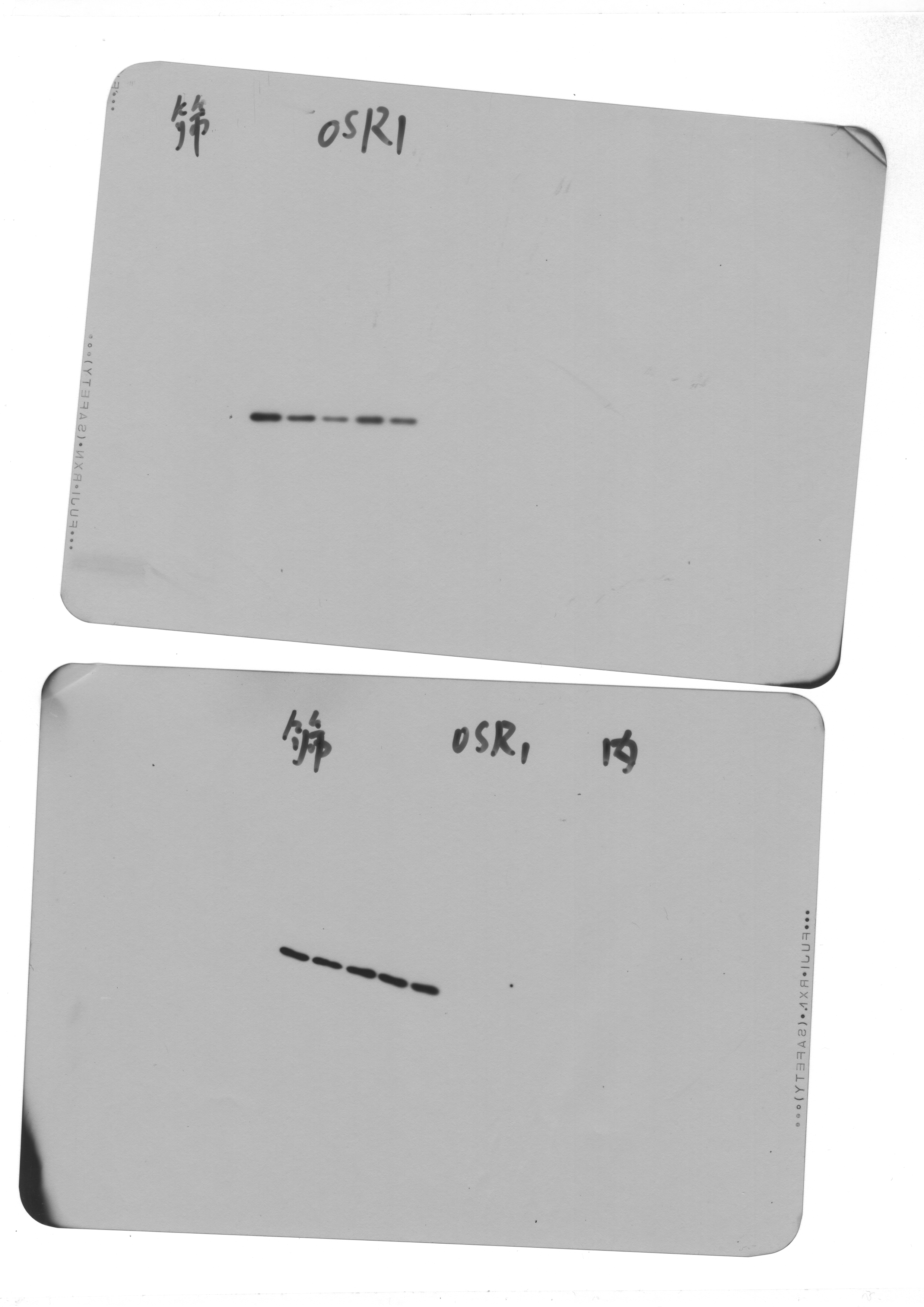


Figure 2D

OSR1

From left to right ： SK-control；SK-vector；SK-oeOSR1


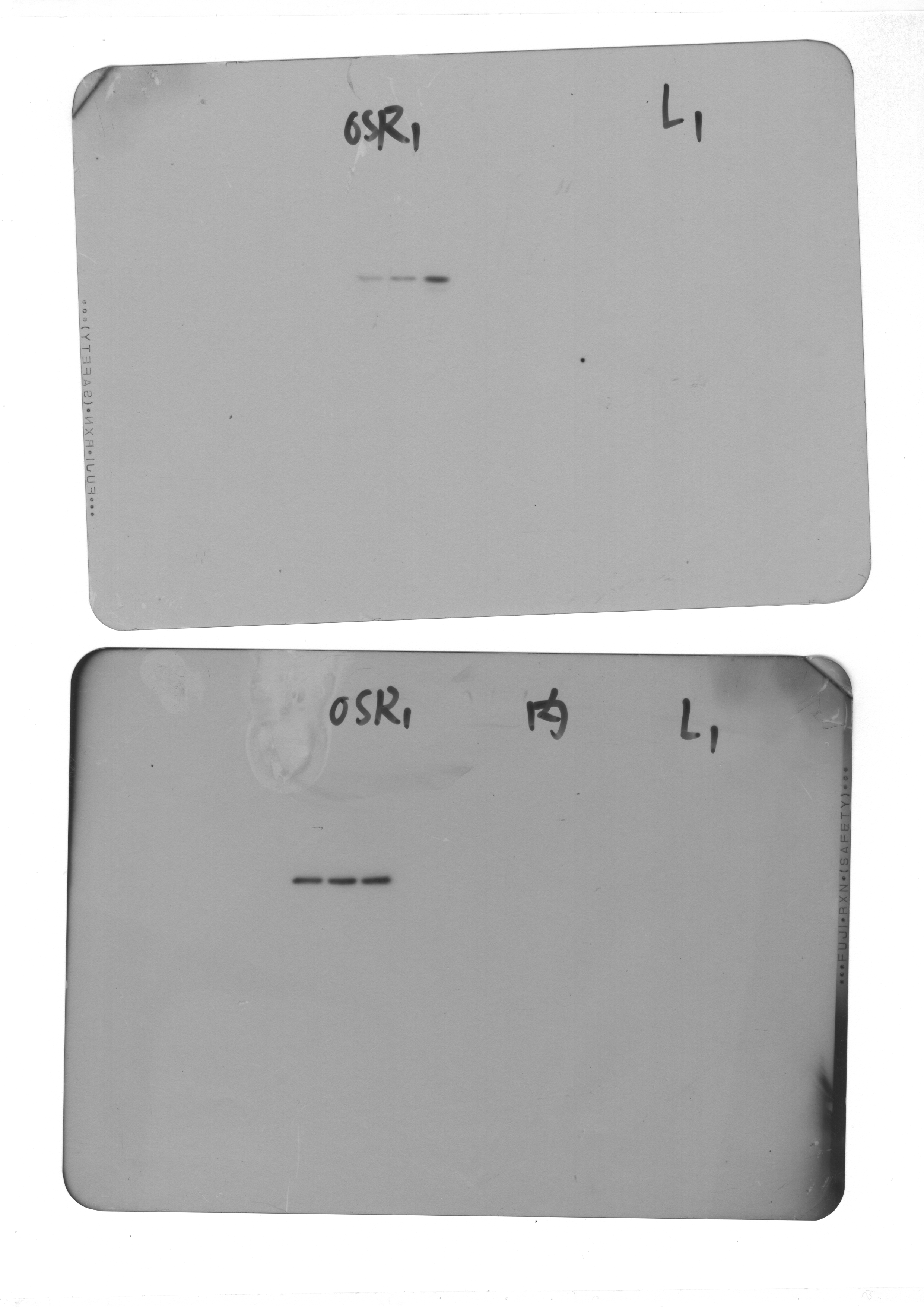


β-actin

From left to right ： SK-control；SK-vector；SK-oeOSR1


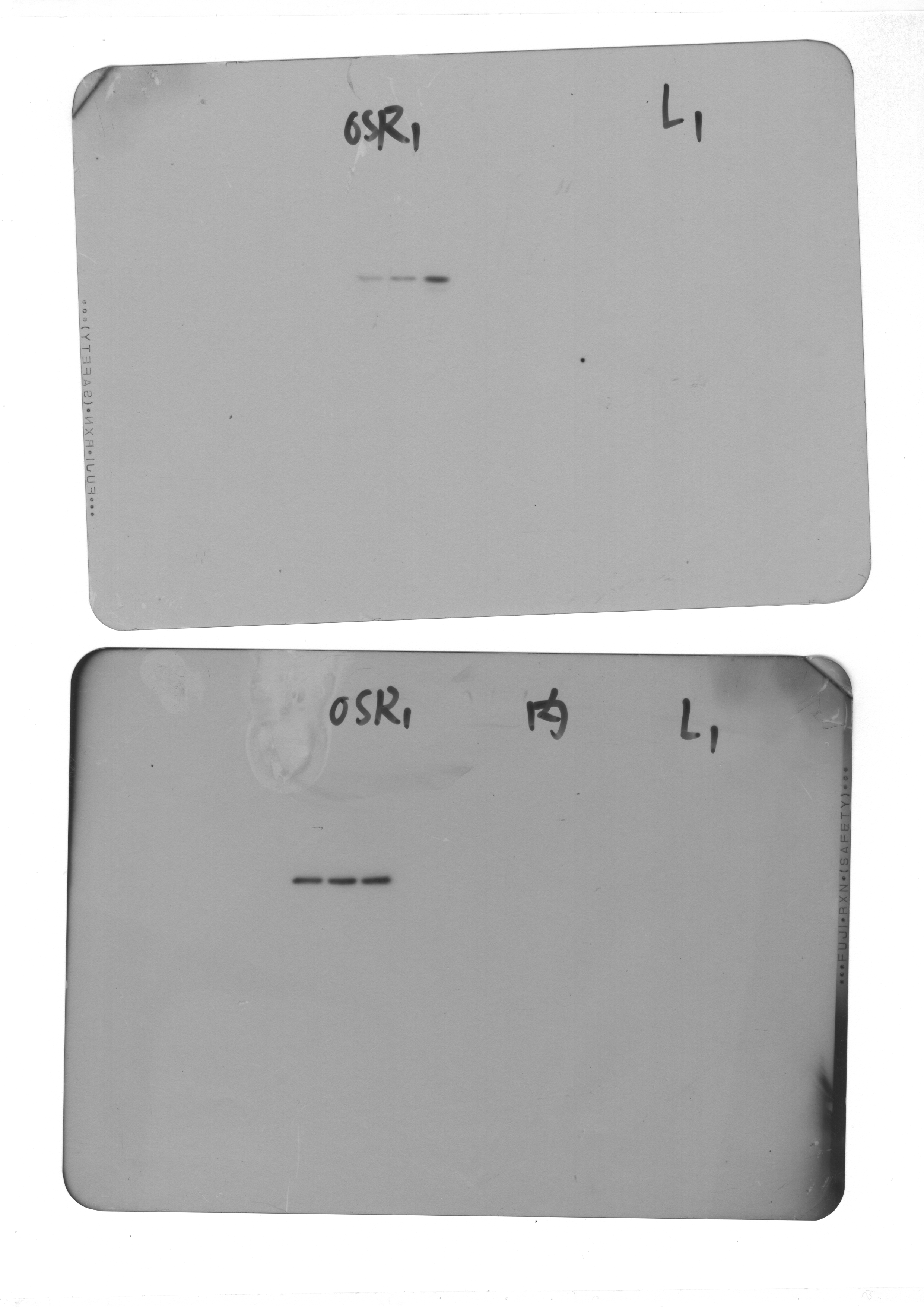


OSR1

From left to right ： OV-control；OV-vector；OV-oeOSR1


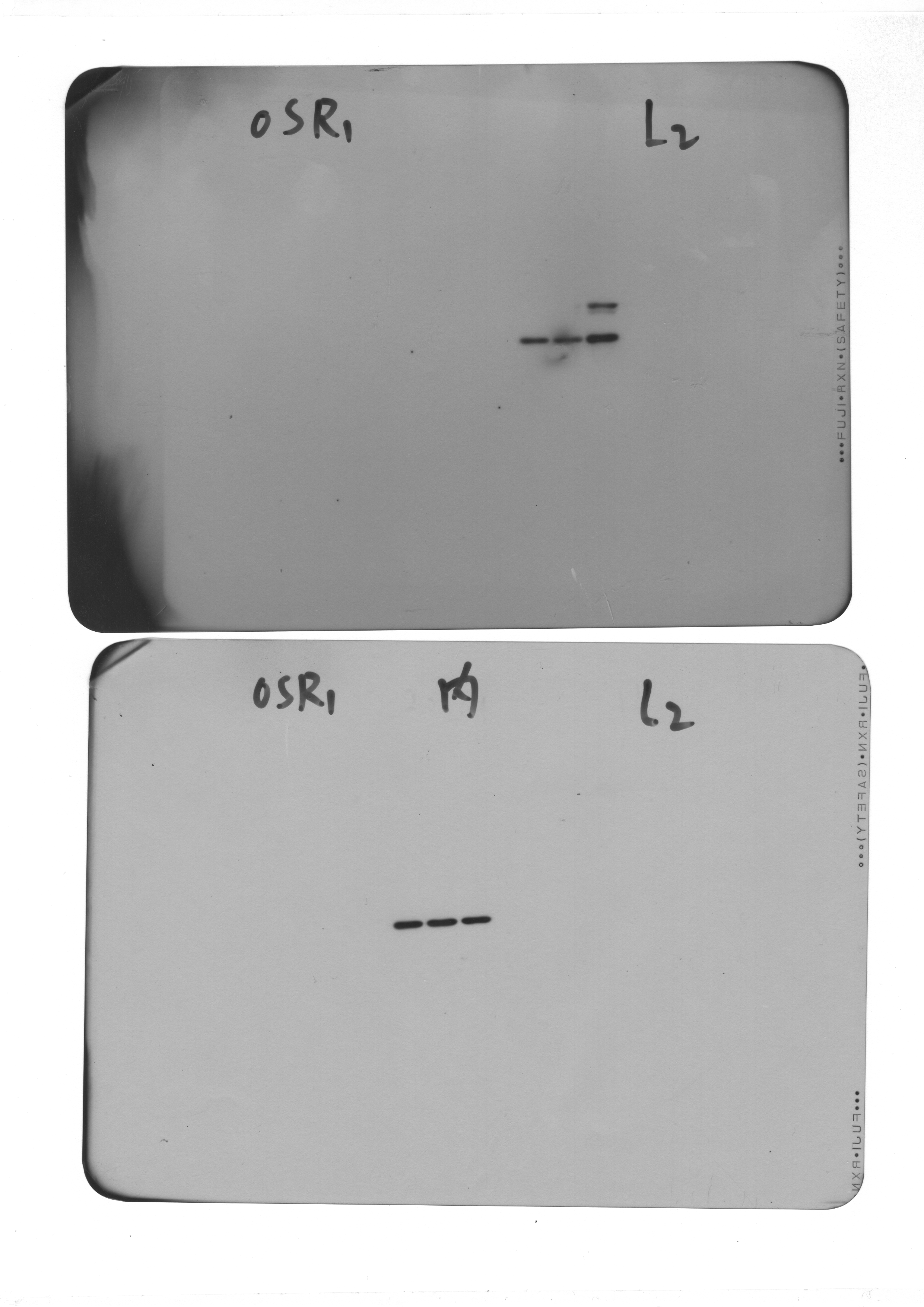


β-actin

From left to right ： OV-control；OV-vector；OV-oeOSR1


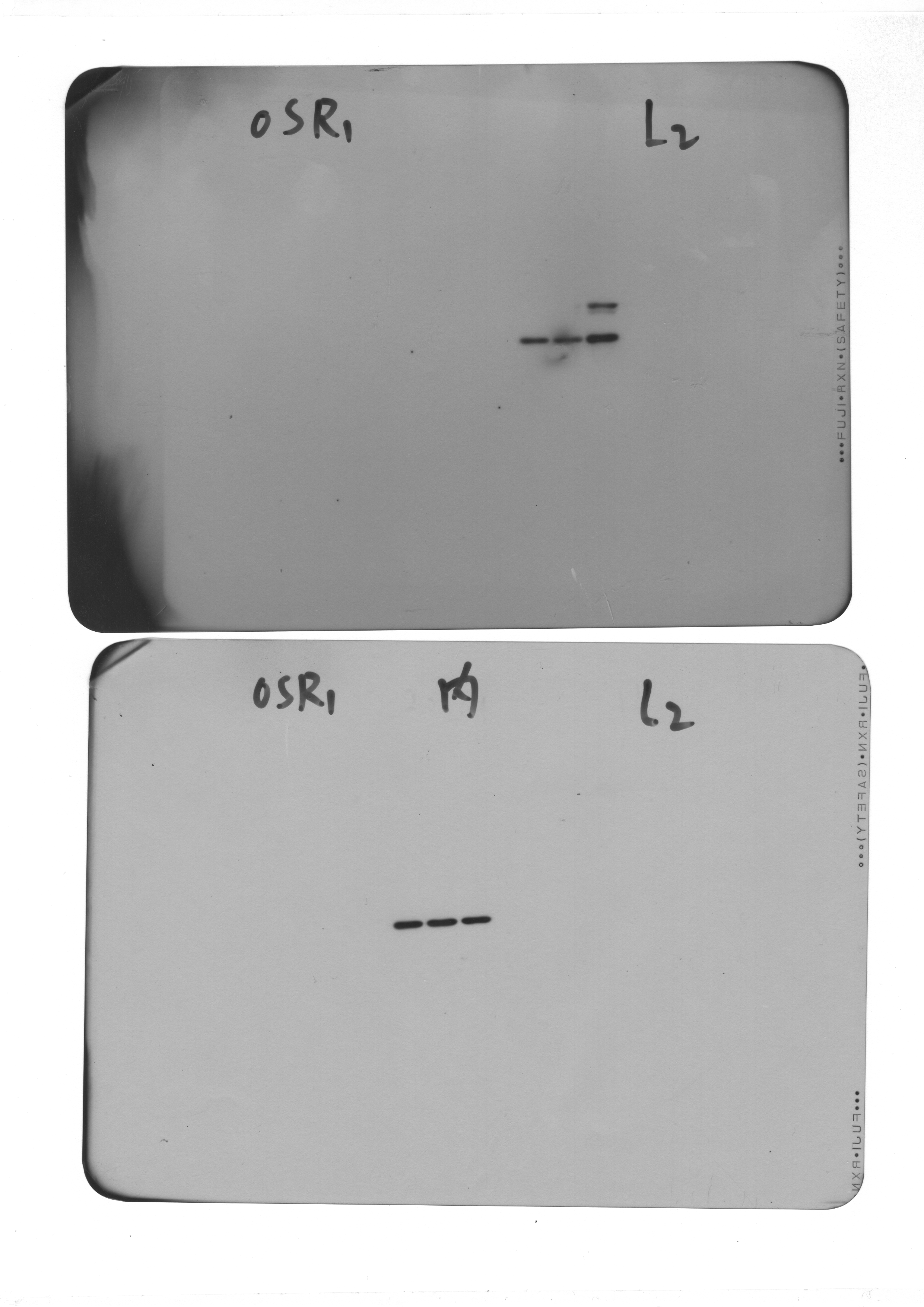


Figure 2F

OSR1

From left to right ： control；si-NC；OSR1-siRNA-1;OSR1-siRNA-2;OSR1-siRNA-3;


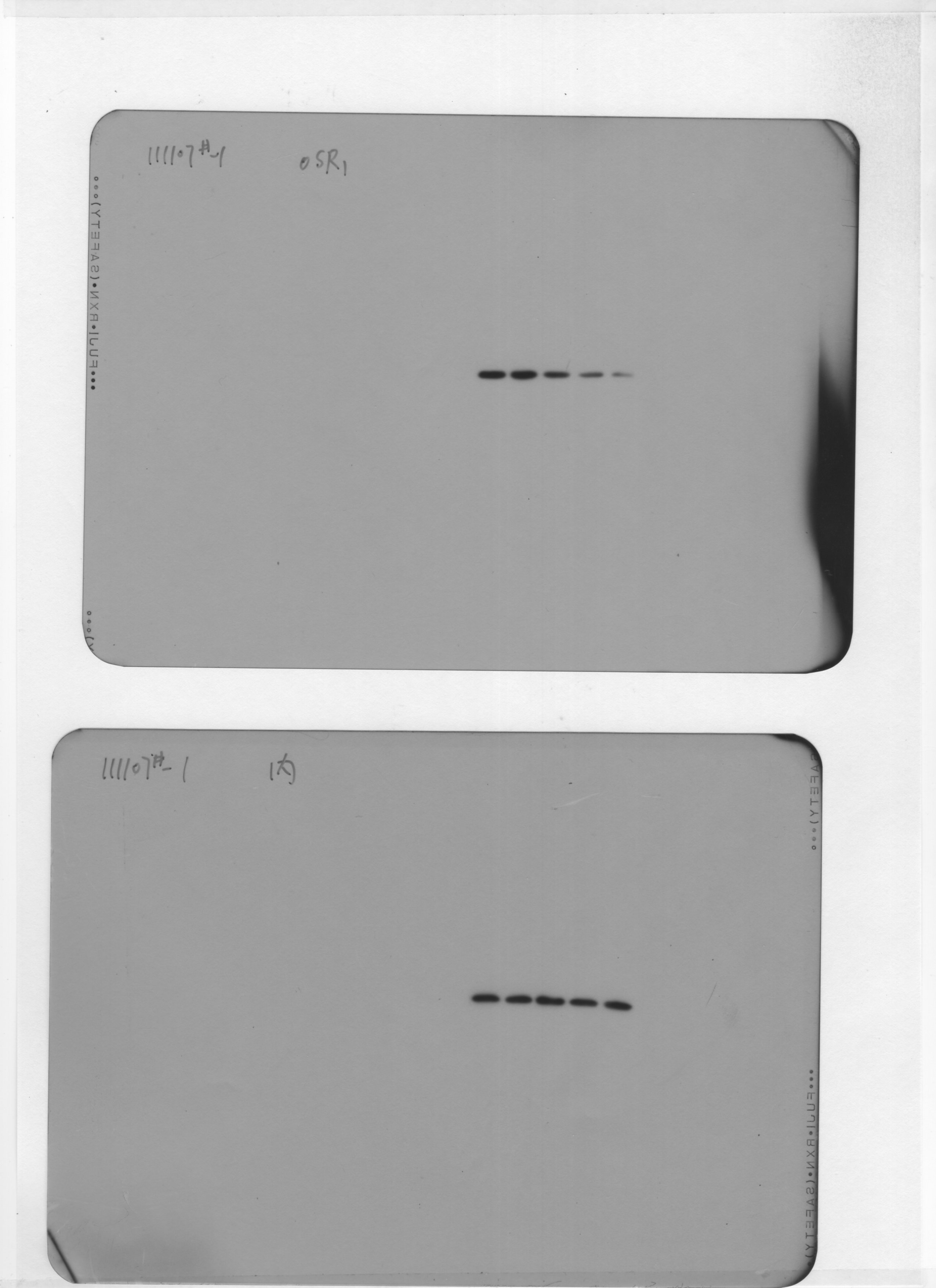


β-actin

From left to right ： control；si-NC；OSR1-siRNA-1;OSR1-siRNA-2;OSR1-siRNA-3;


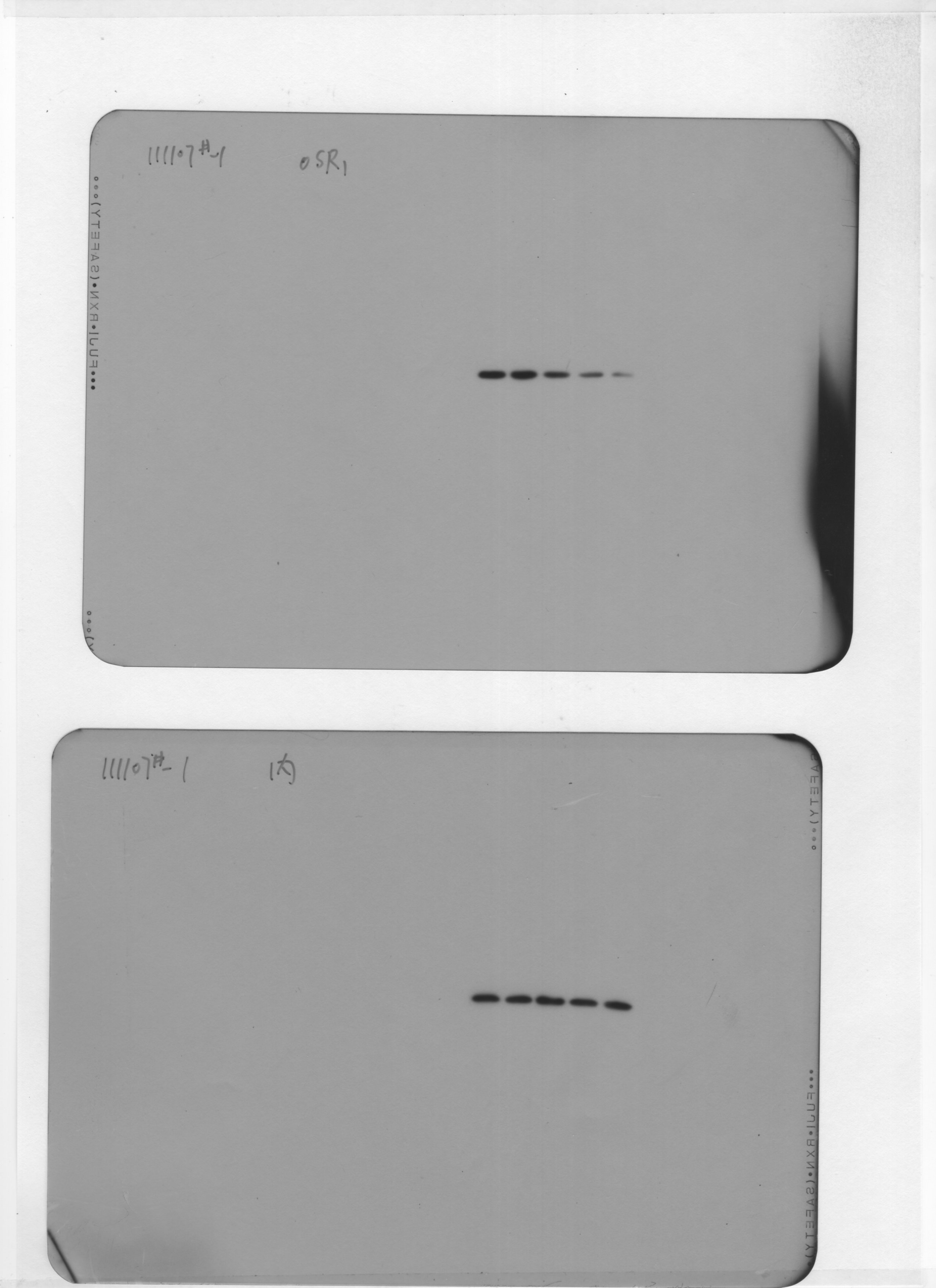


Figure 2G

p-p65

From left to right ： SK-control；SK-vector；SK-OSR1;OV-control；OV-vector；OV-OSR1；


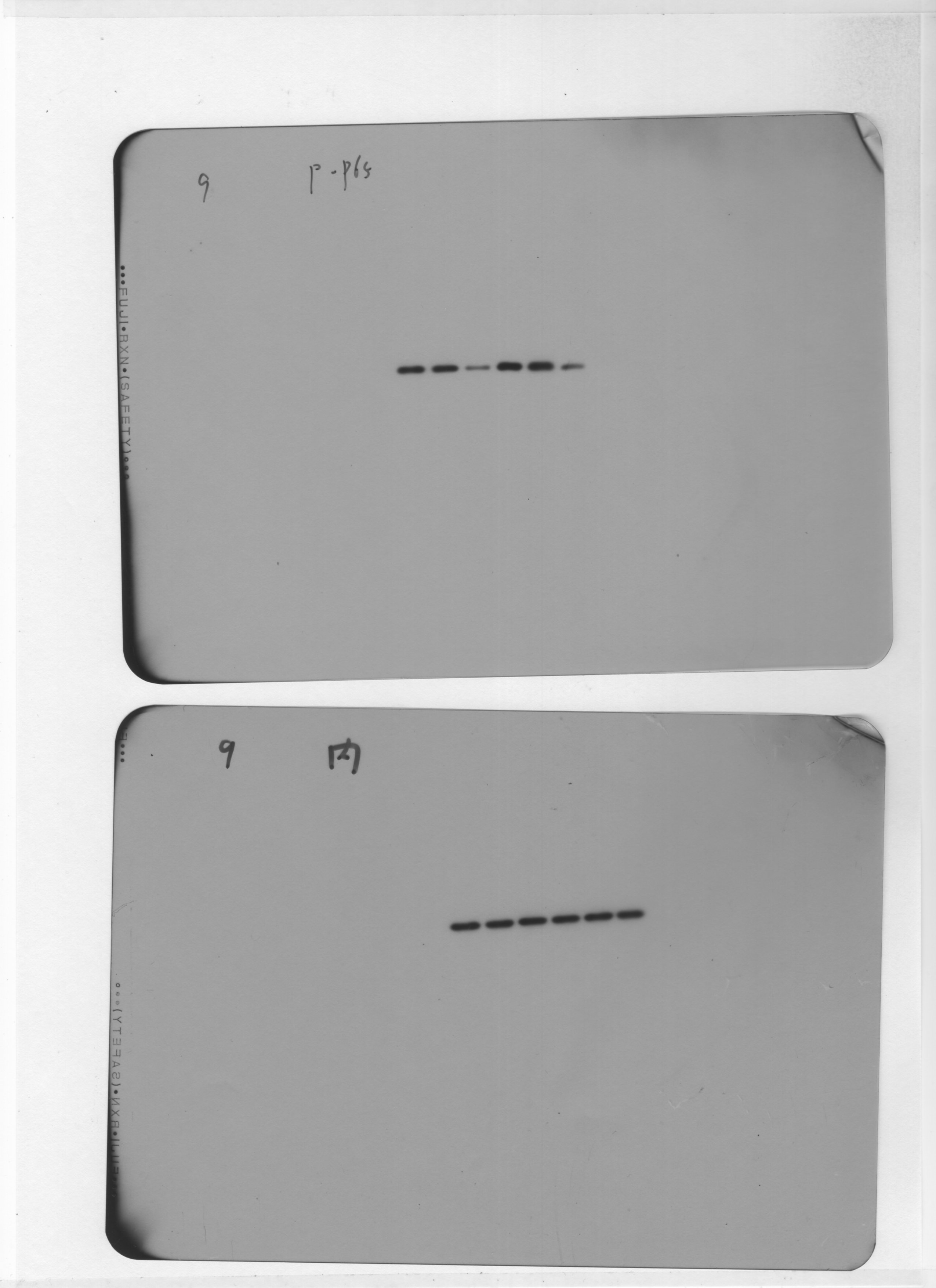


p-65

From left to right ： SK-control；SK-vector；SK-OSR1;OV-control；OV-vector；OV-OSR1；


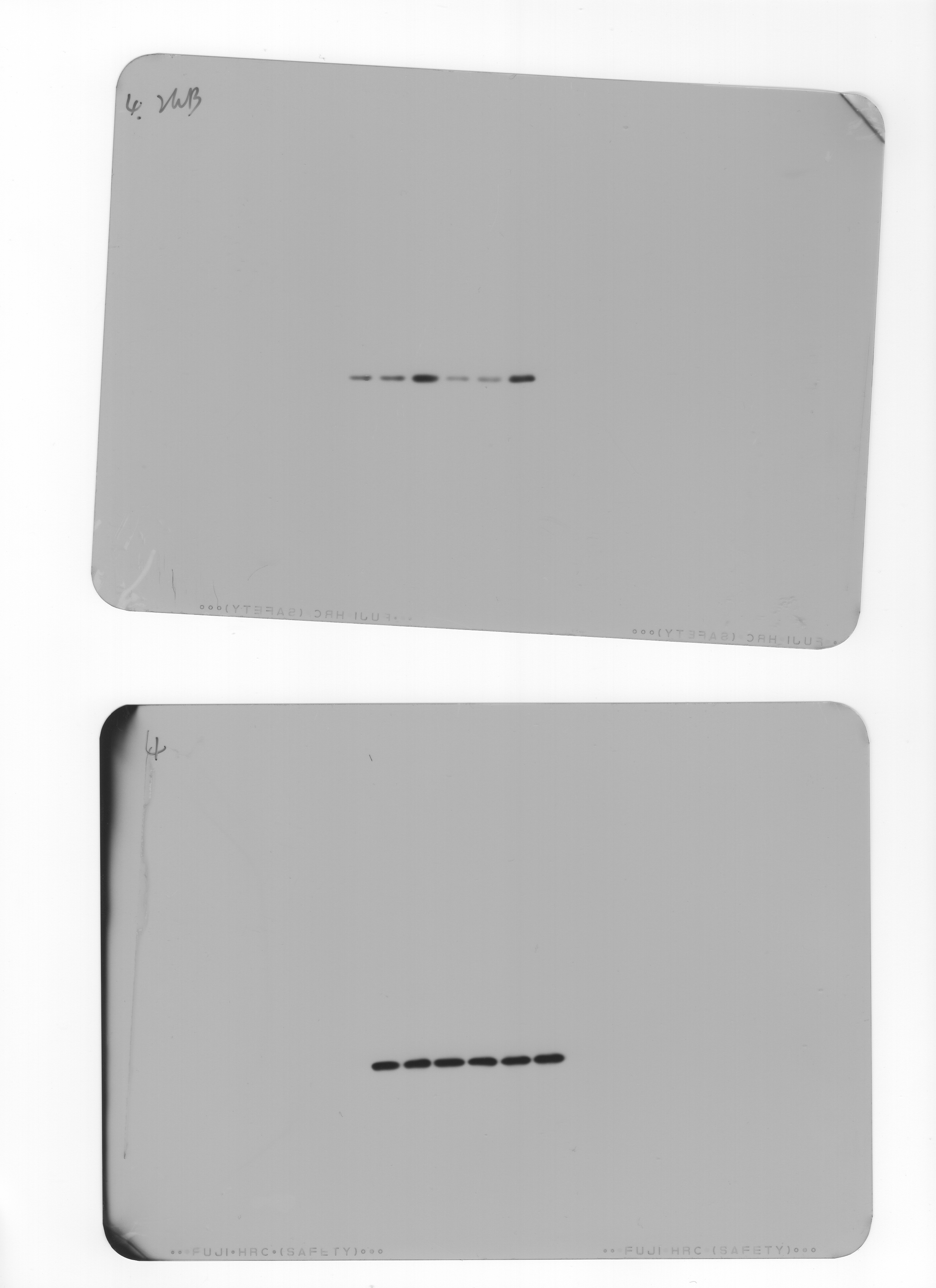


β-actin

From left to right ： SK-control；SK-vector；SK-OSR1;OV-control；OV-vector；OV-OSR1；


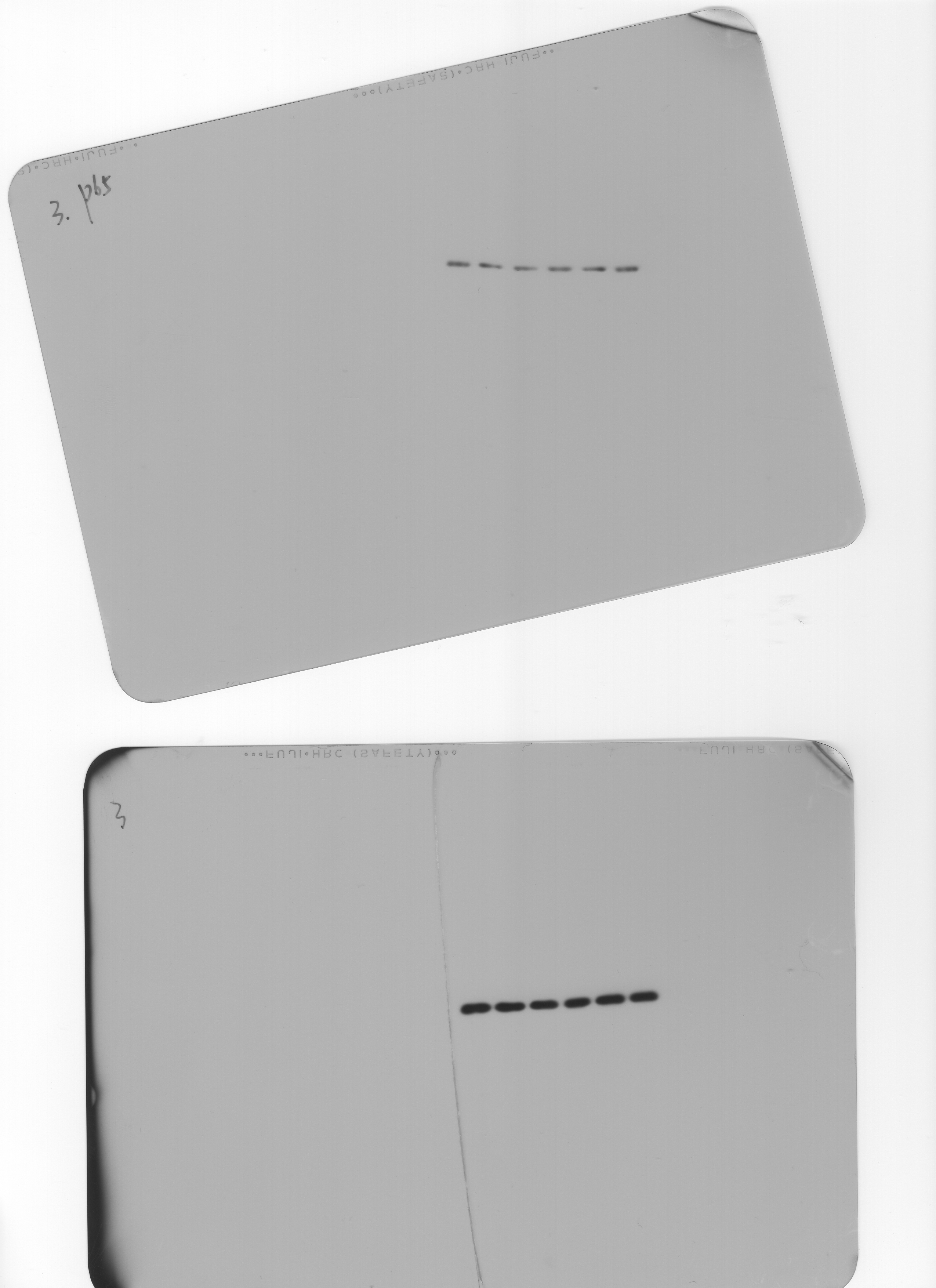


p-IκBα

From left to right ： SK-control；SK-vector；SK-OSR1;OV-control；OV-vector；OV-OSR1；


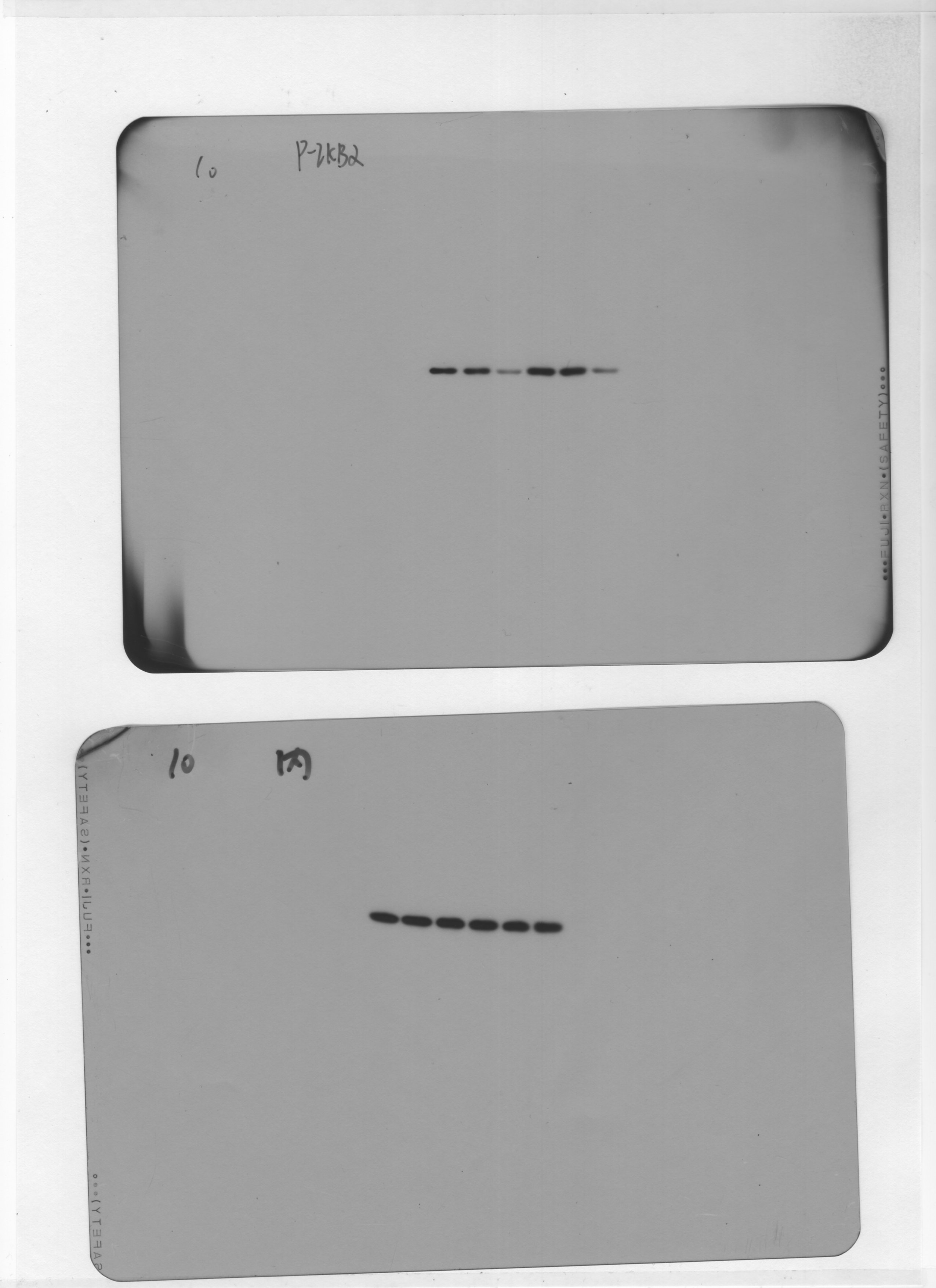


IκBα

From left to right ： SK-control；SK-vector；SK-OSR1;OV-control；OV-vector；OV-OSR1；


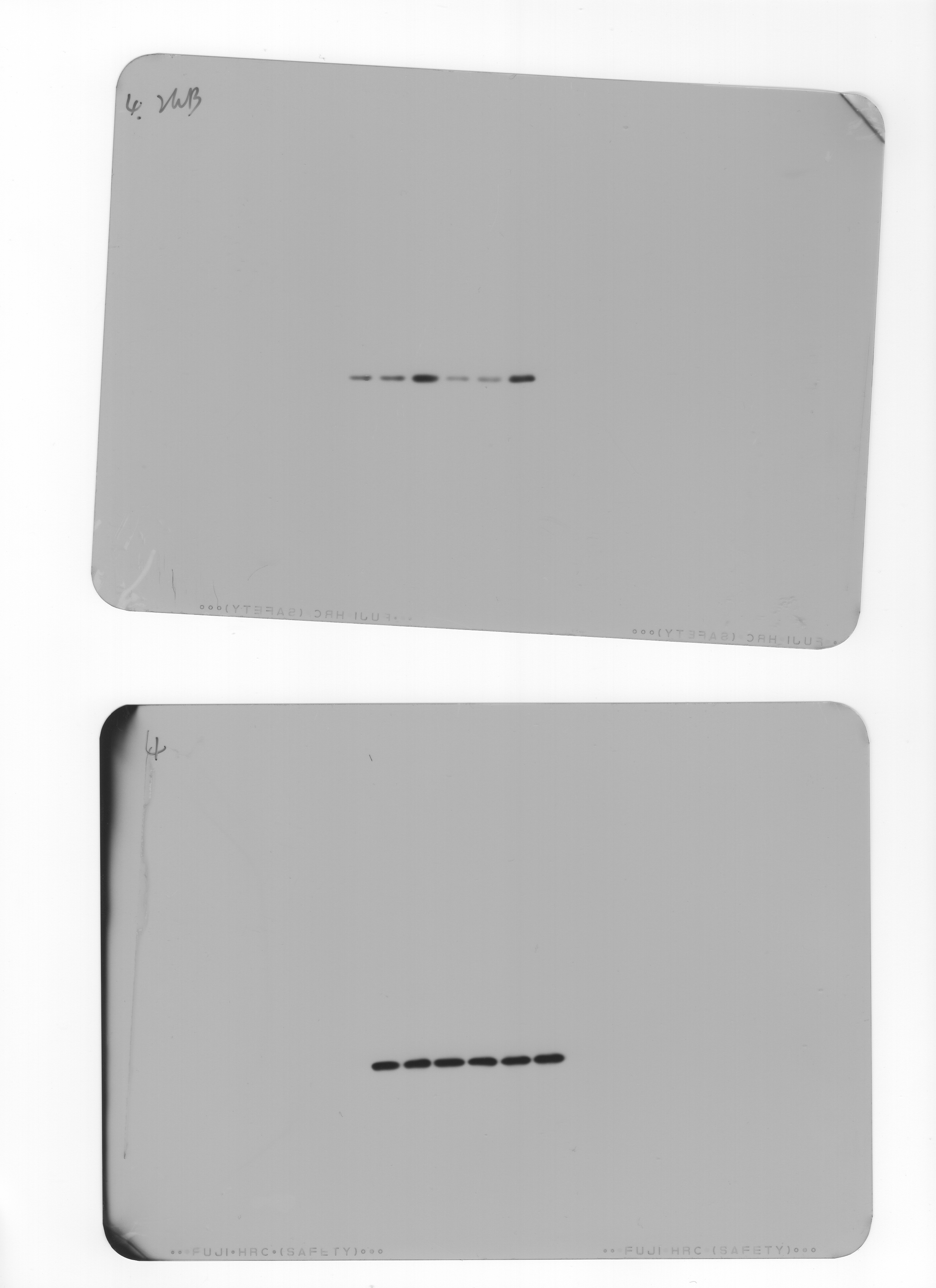


β-actin

From left to right ： SK-control；SK-vector；SK-OSR1;OV-control；OV-vector；OV-OSR1；


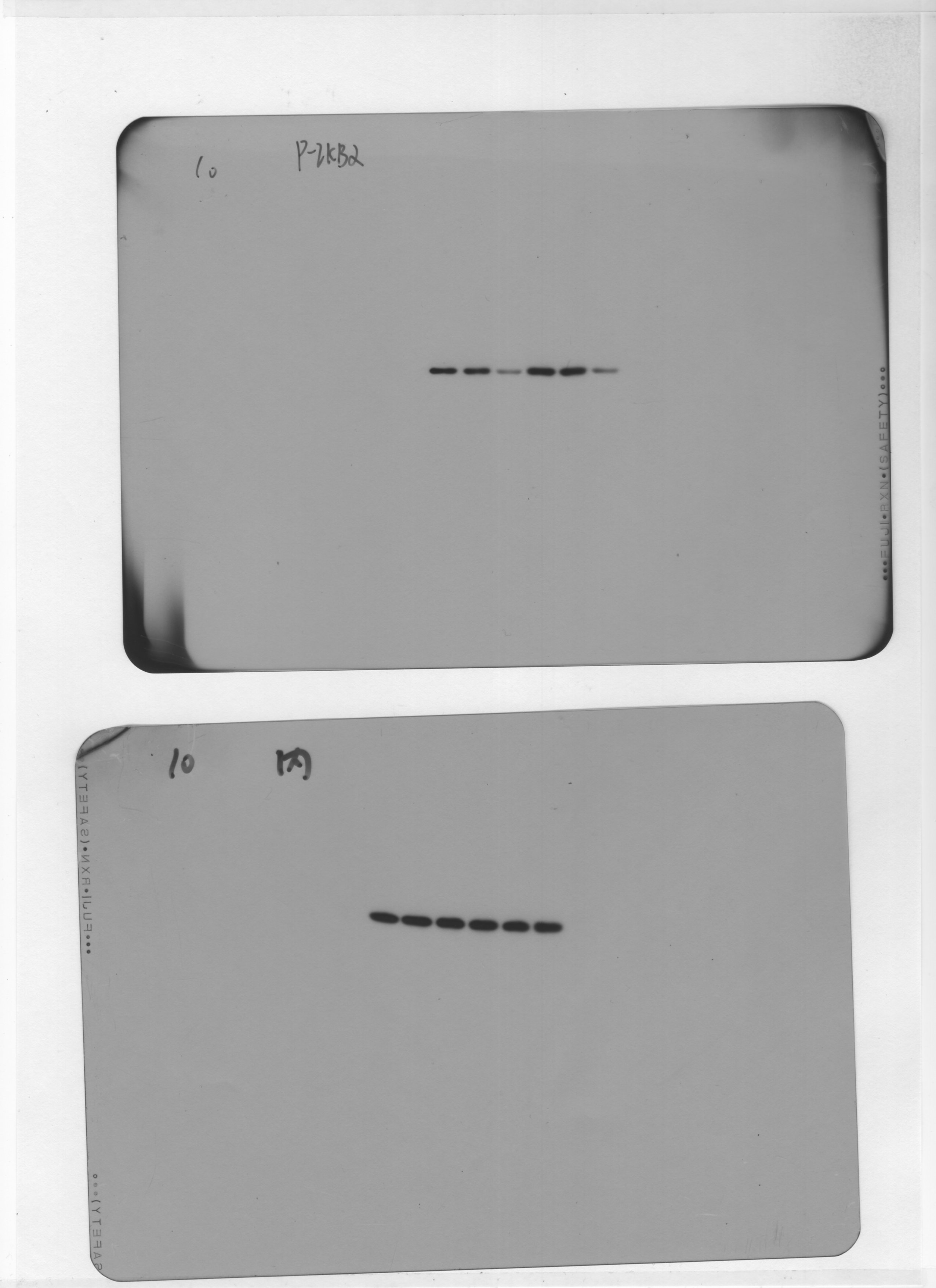


Figure 2H

p-p65

From left to right ： control；si-NC；OSR1-siRNA-3；the fourth is an unrelated group；


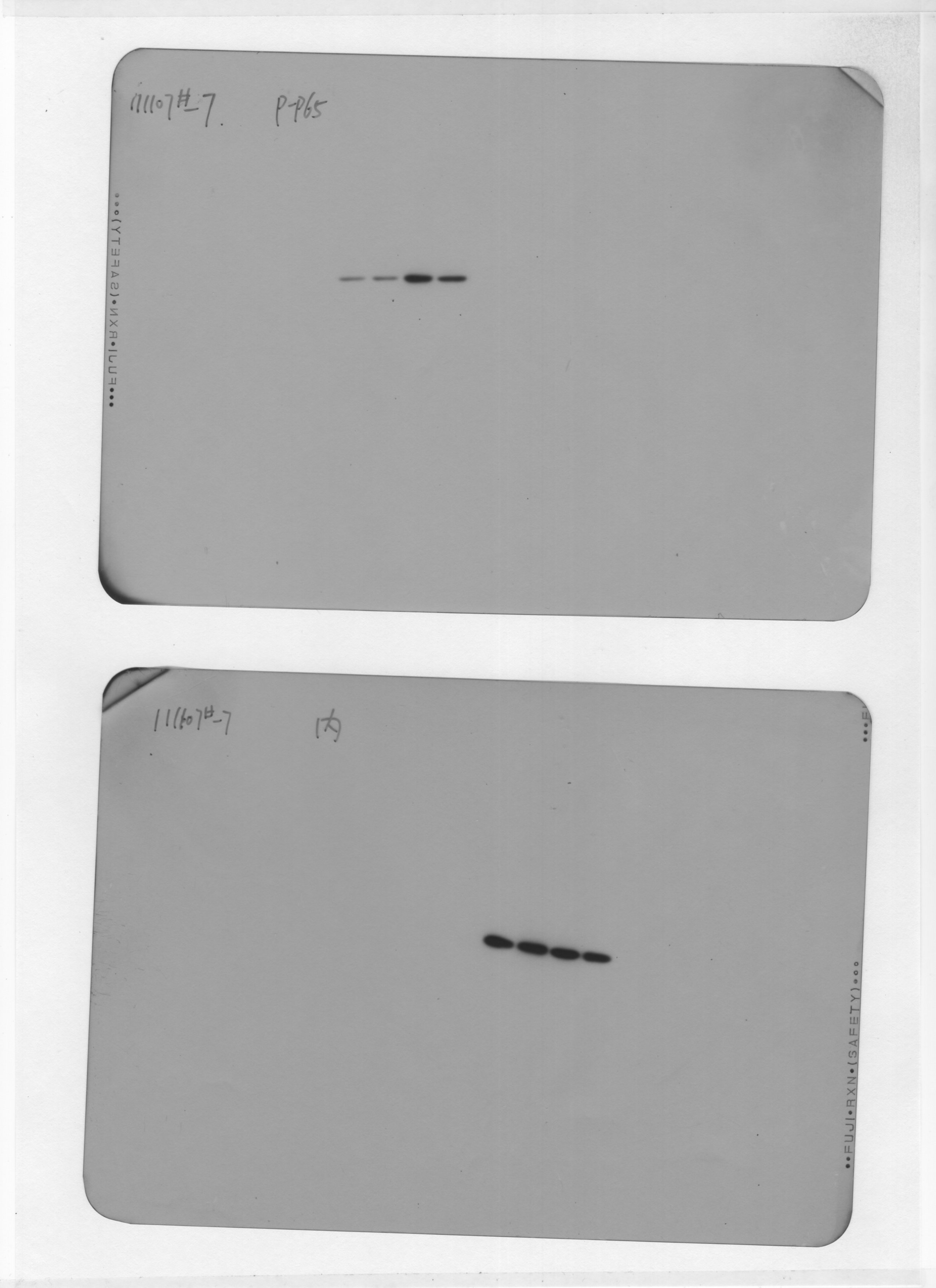


p65

From left to right ： control；si-NC；OSR1-siRNA-3；the fourth is an unrelated group；


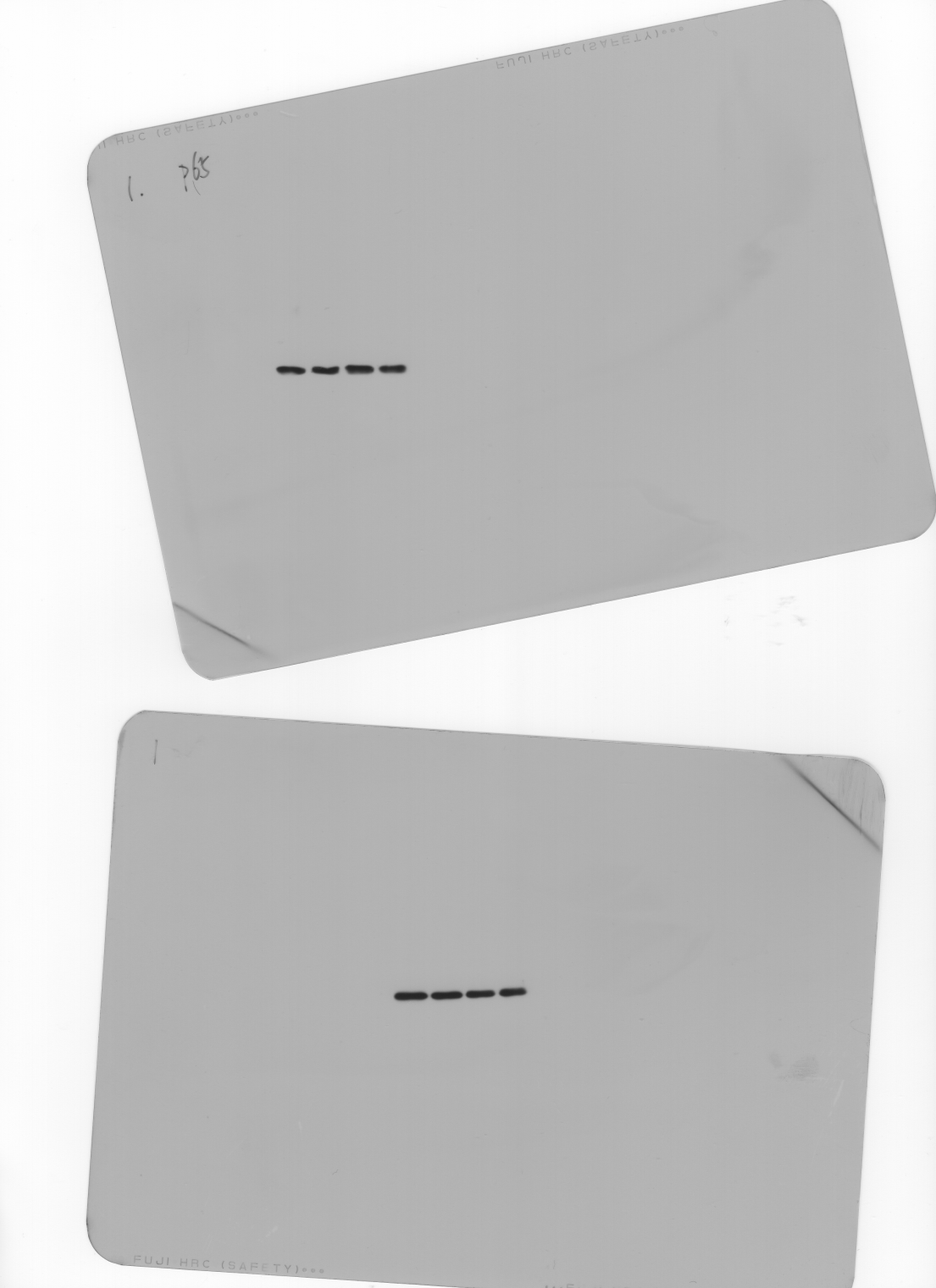


β-actin

From left to right ：control；si-NC；OSR1-siRNA-3；the fourth is an unrelated group；


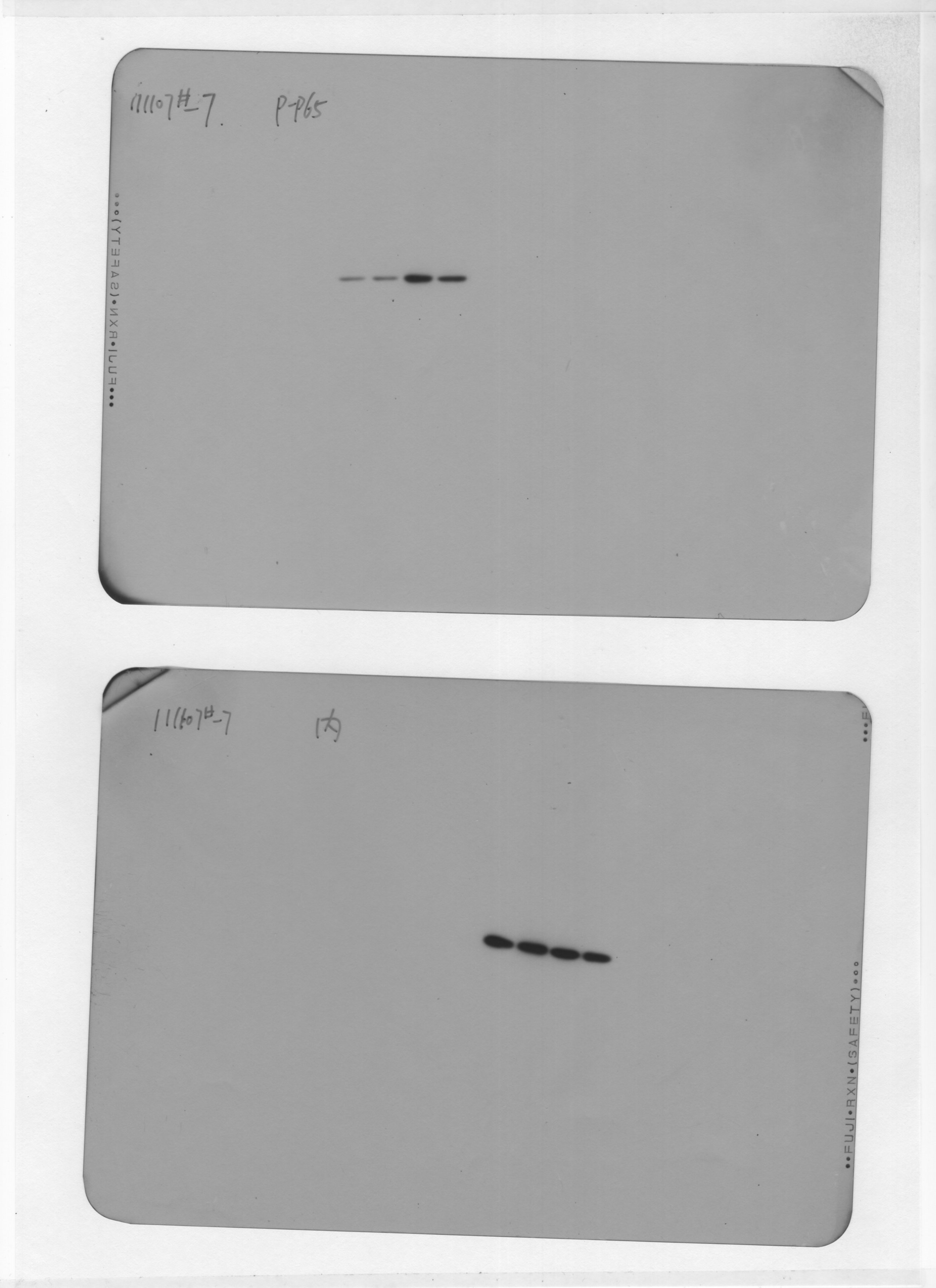


p-IκBα

From left to right ：control；si-NC；OSR1-siRNA-3；the fourth is an unrelated group；


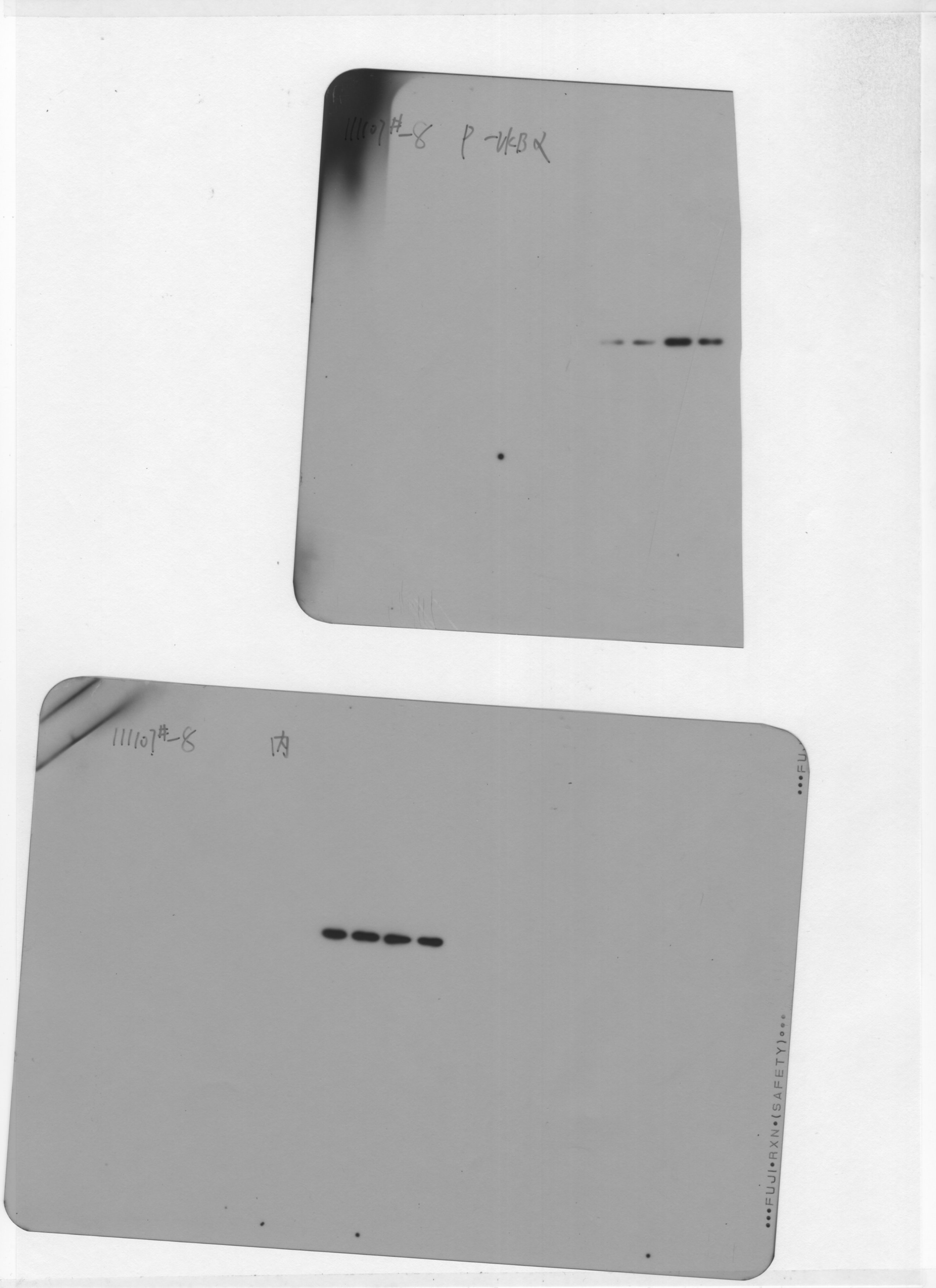


IκBα

From left to right ：control；si-NC；OSR1-siRNA-3；the fourth is an unrelated group；


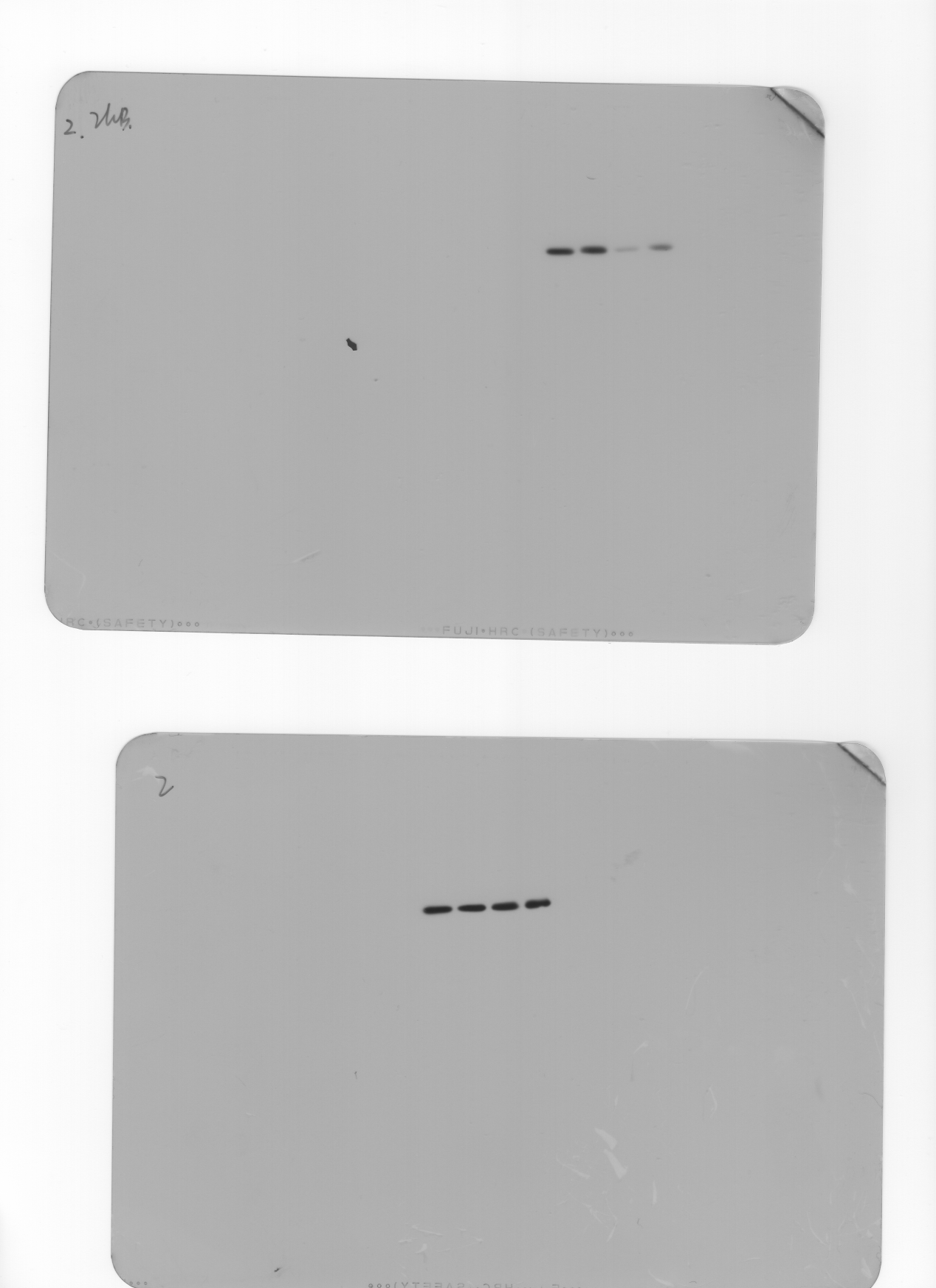


β-actin

From left to right ：control；si-NC；OSR1-siRNA-3；the fourth is an unrelated group；


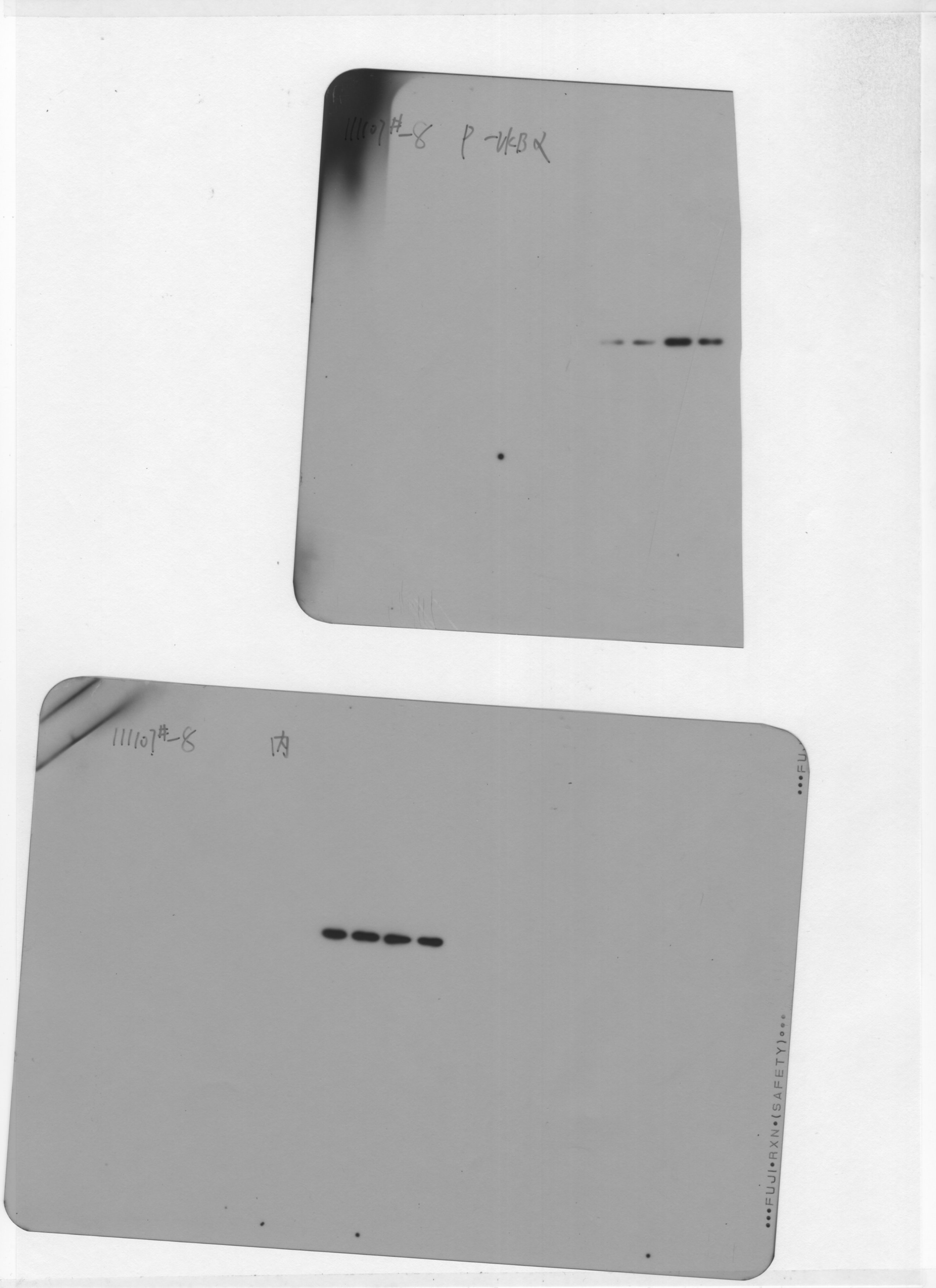


Figure 3C

PCNA

From left to right ：control；si-NC；OSR1-siRNA-3；OSR1-siRNA-3+BAY 11-7082；


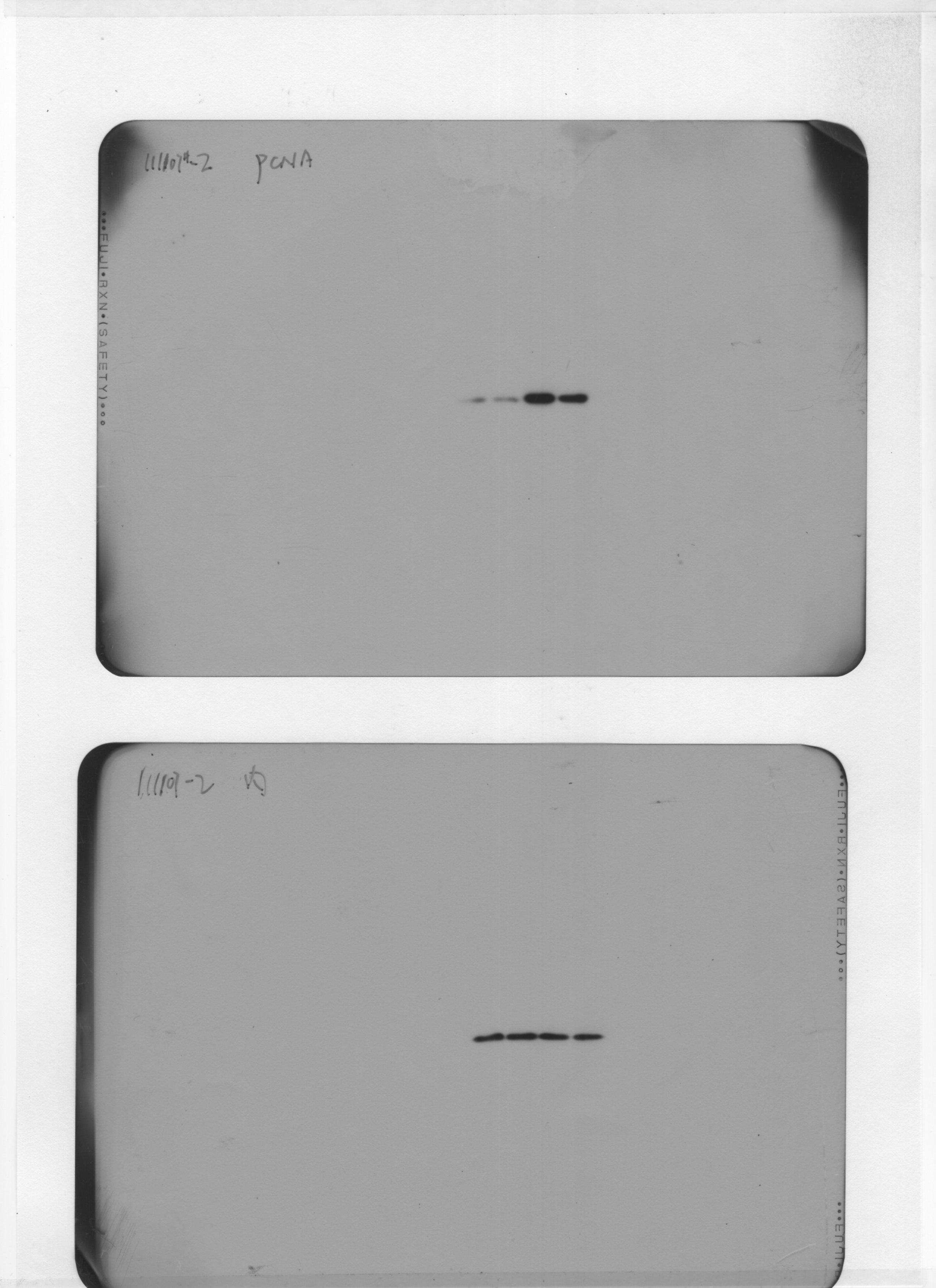


β-actin

From left to right ：control；si-NC；OSR1-siRNA-3；OSR1-siRNA-3+BAY 11-7082；


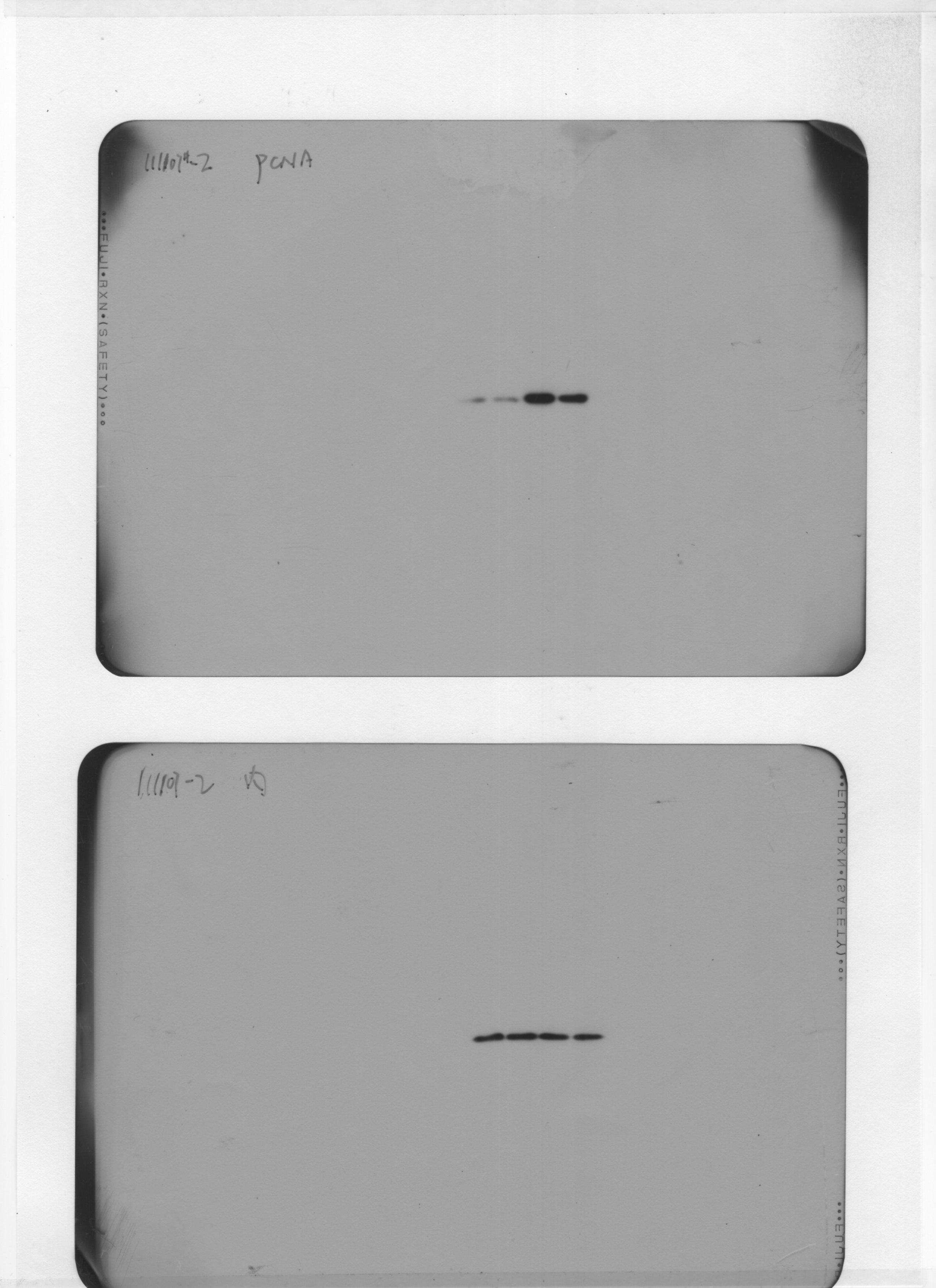


cyclinD1

From left to right ：control；si-NC；OSR1-siRNA-3；OSR1-siRNA-3+BAY 11-7082；


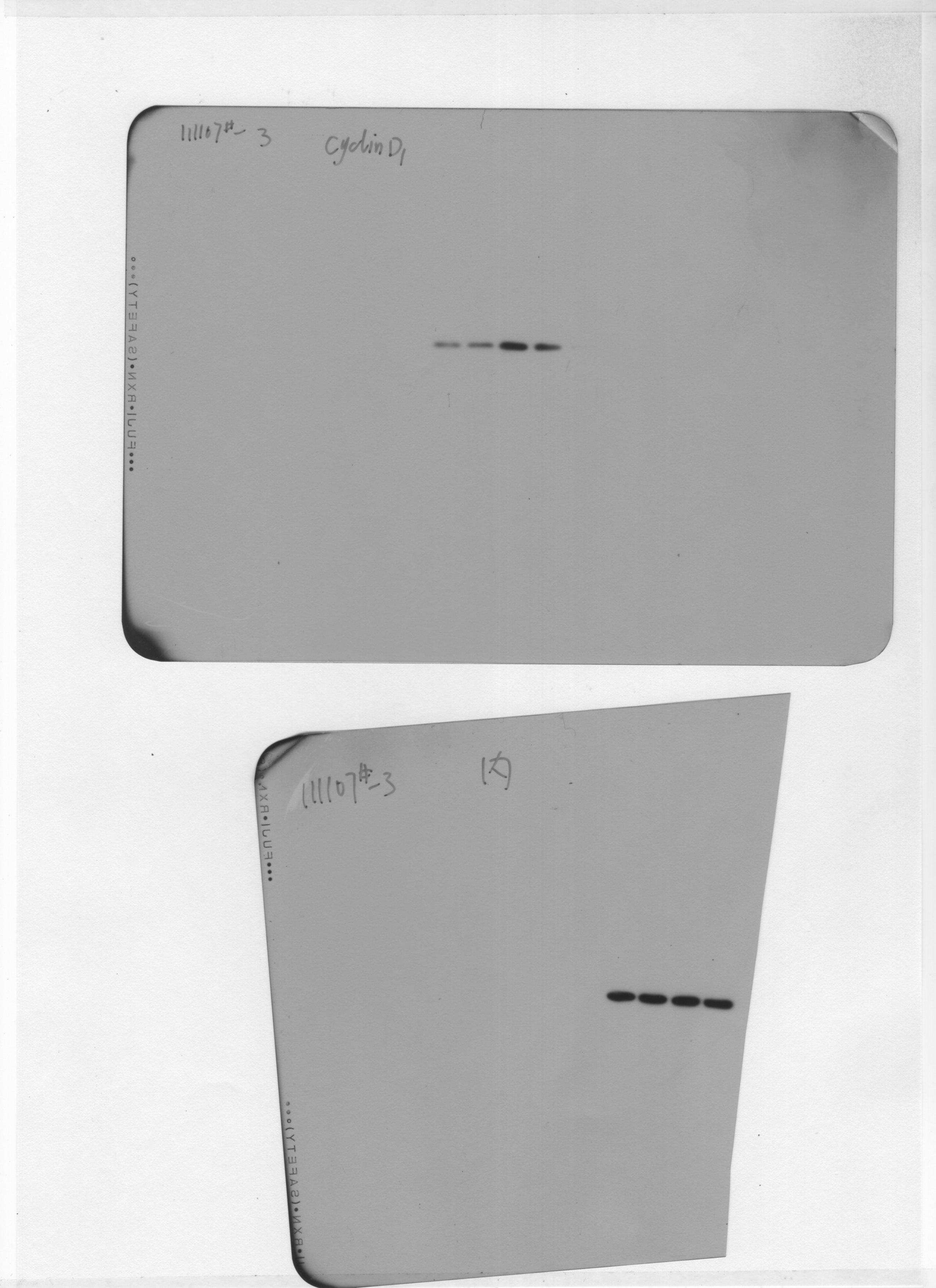


βactin

From left to right ：control；si-NC；OSR1-siRNA-3；OSR1-siRNA-3+BAY 11-7082；


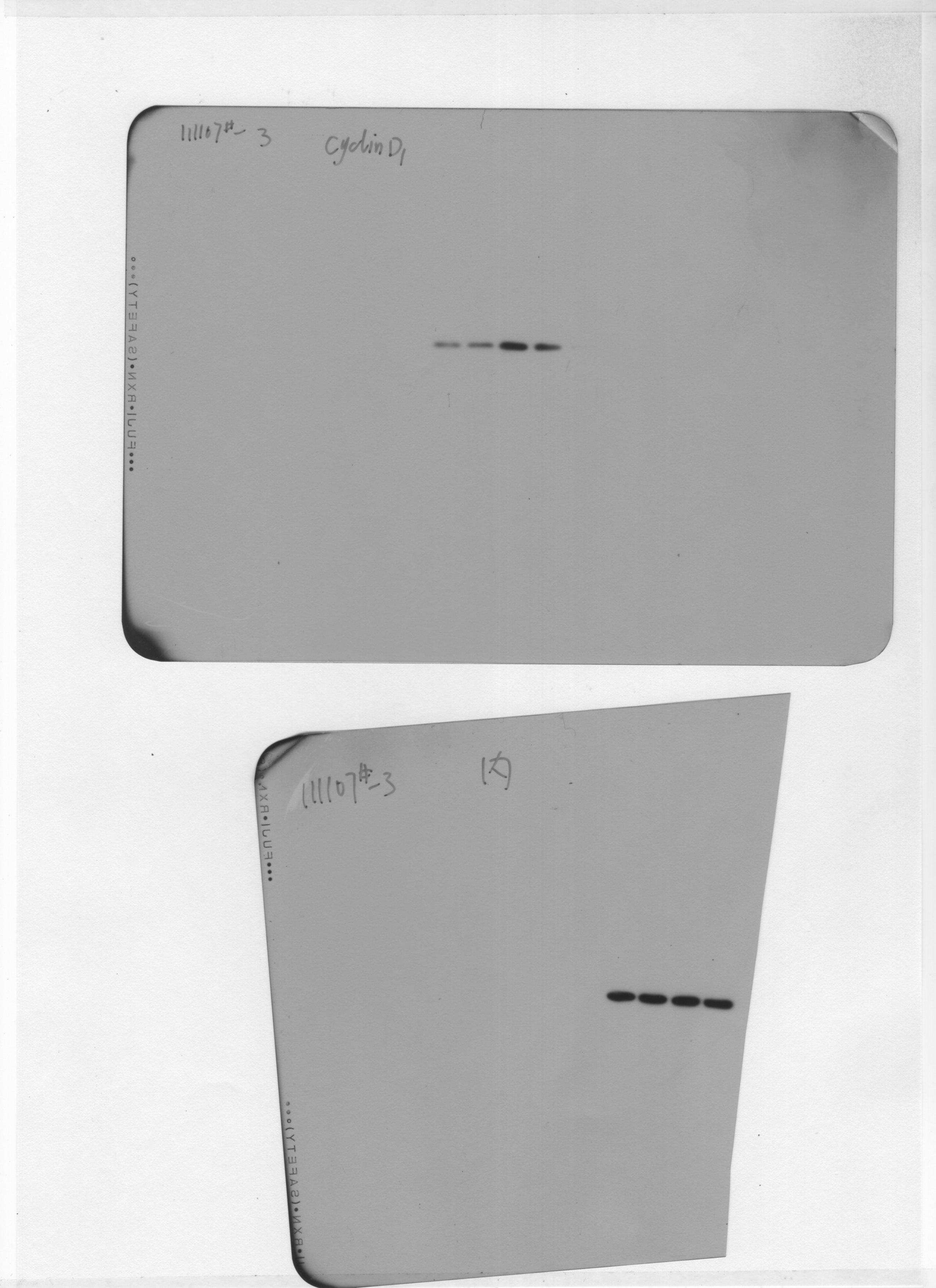


Figure 5C

Bcl-2

From left to right ：control；si-NC；OSR1-siRNA-3；OSR1-siRNA-3+BAY 11-7082；


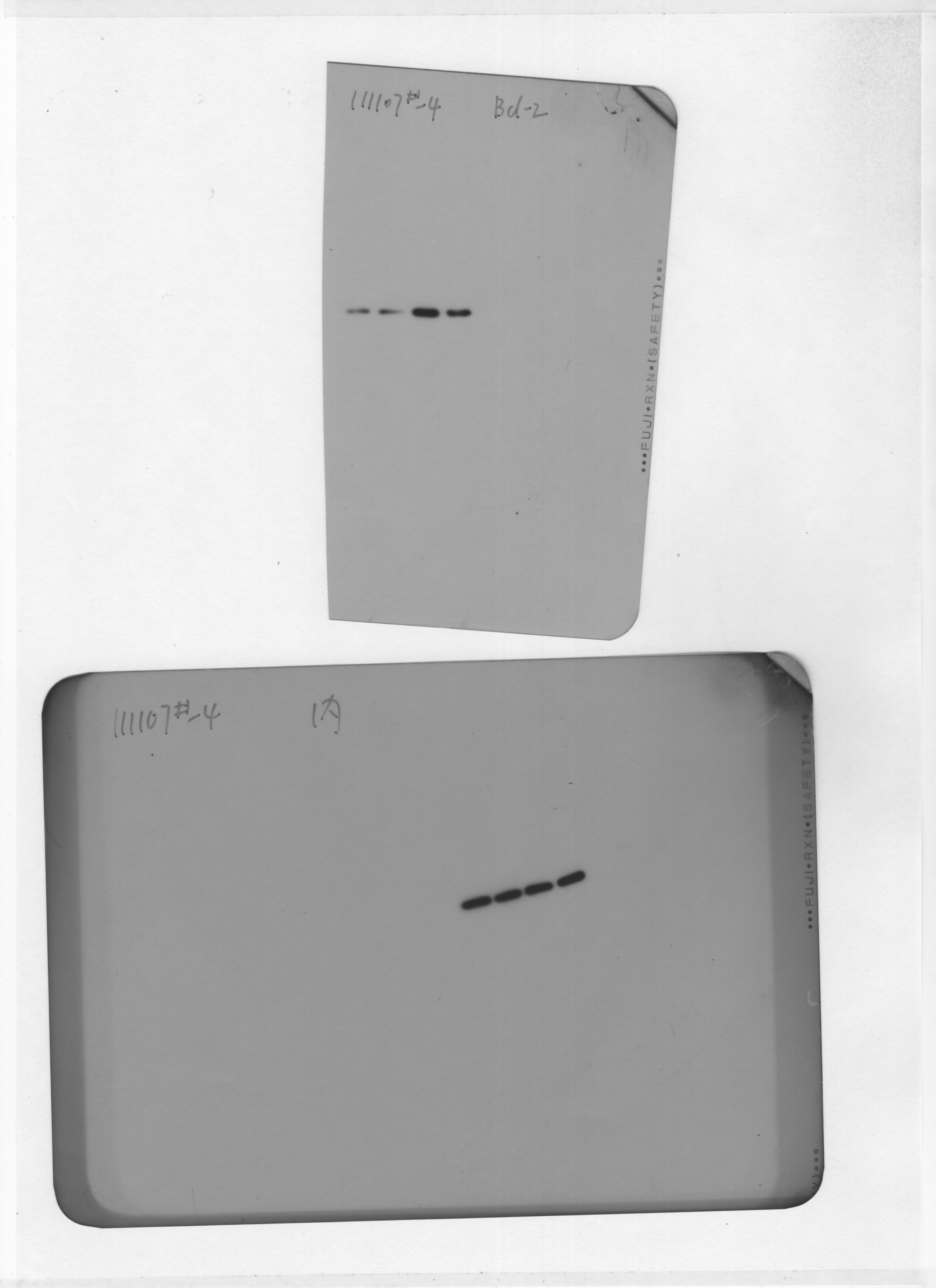


β-actin

From left to right ：control；si-NC；OSR1-siRNA-3；OSR1-siRNA-3+BAY 11-7082；


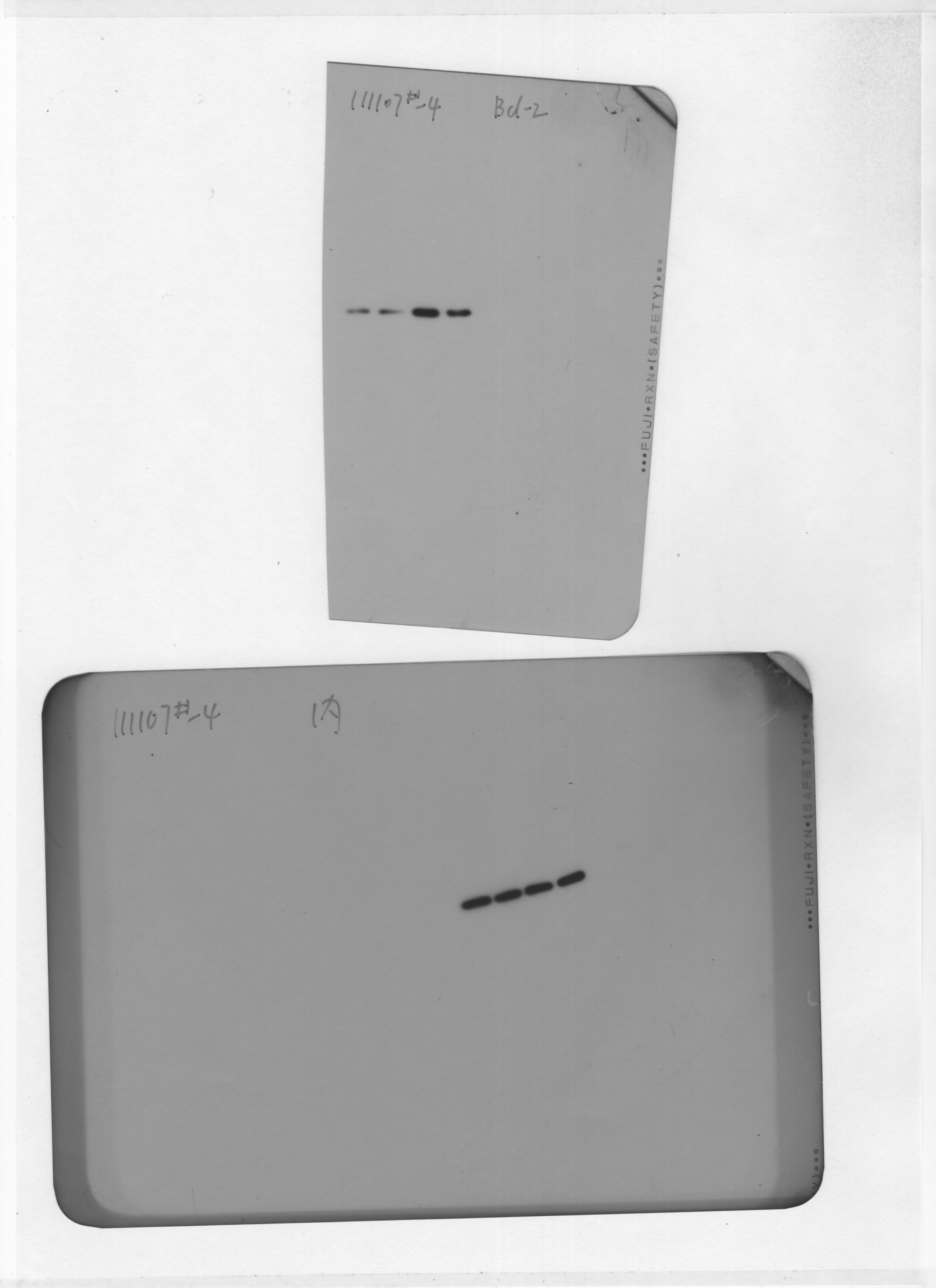


Bax

From left to right ：control；si-NC；OSR1-siRNA-3；OSR1-siRNA-3+BAY 11-7082；


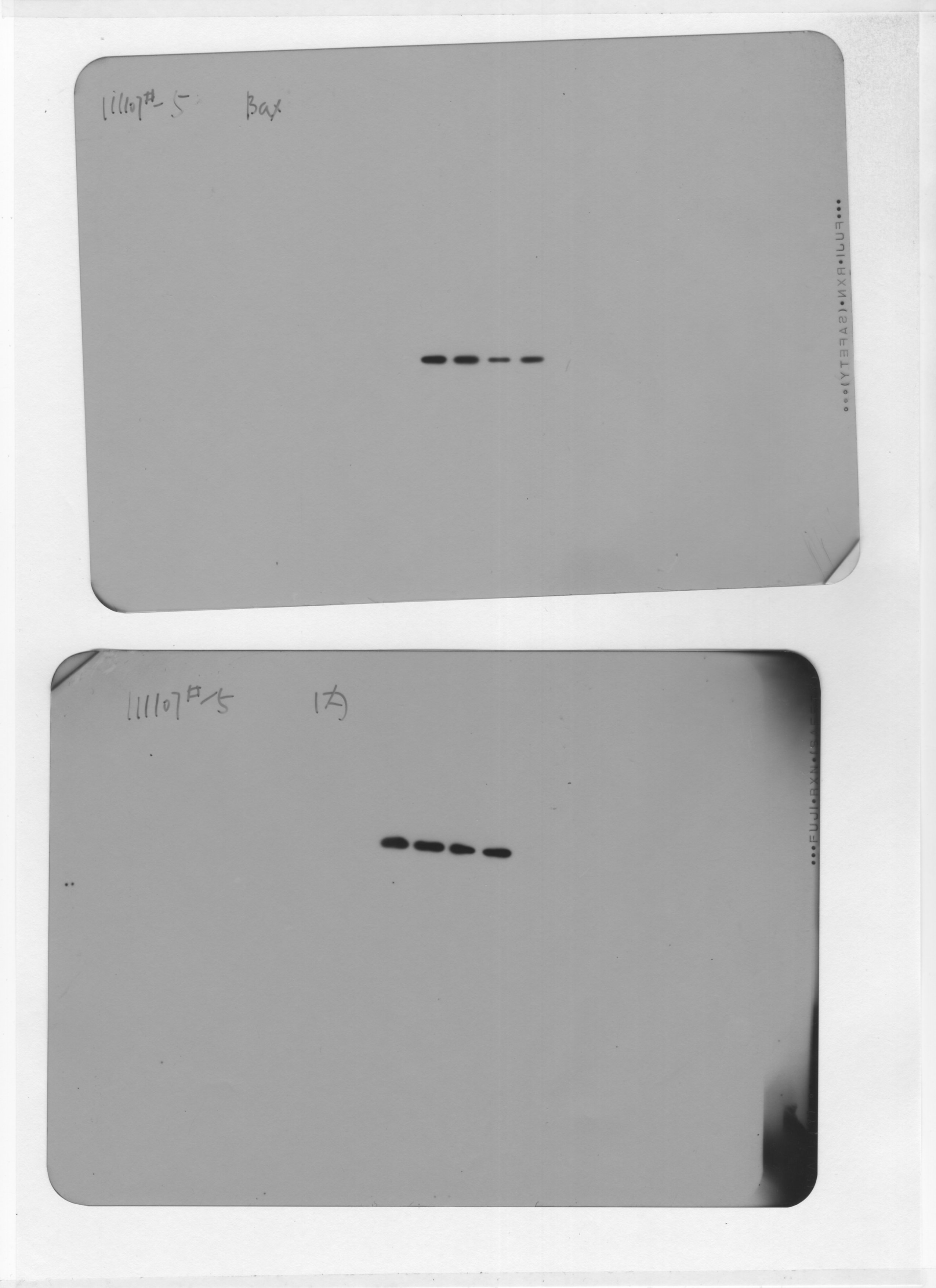


β-actin

From left to right ：control；si-NC；OSR1-siRNA-3；OSR1-siRNA-3+BAY 11-7082；


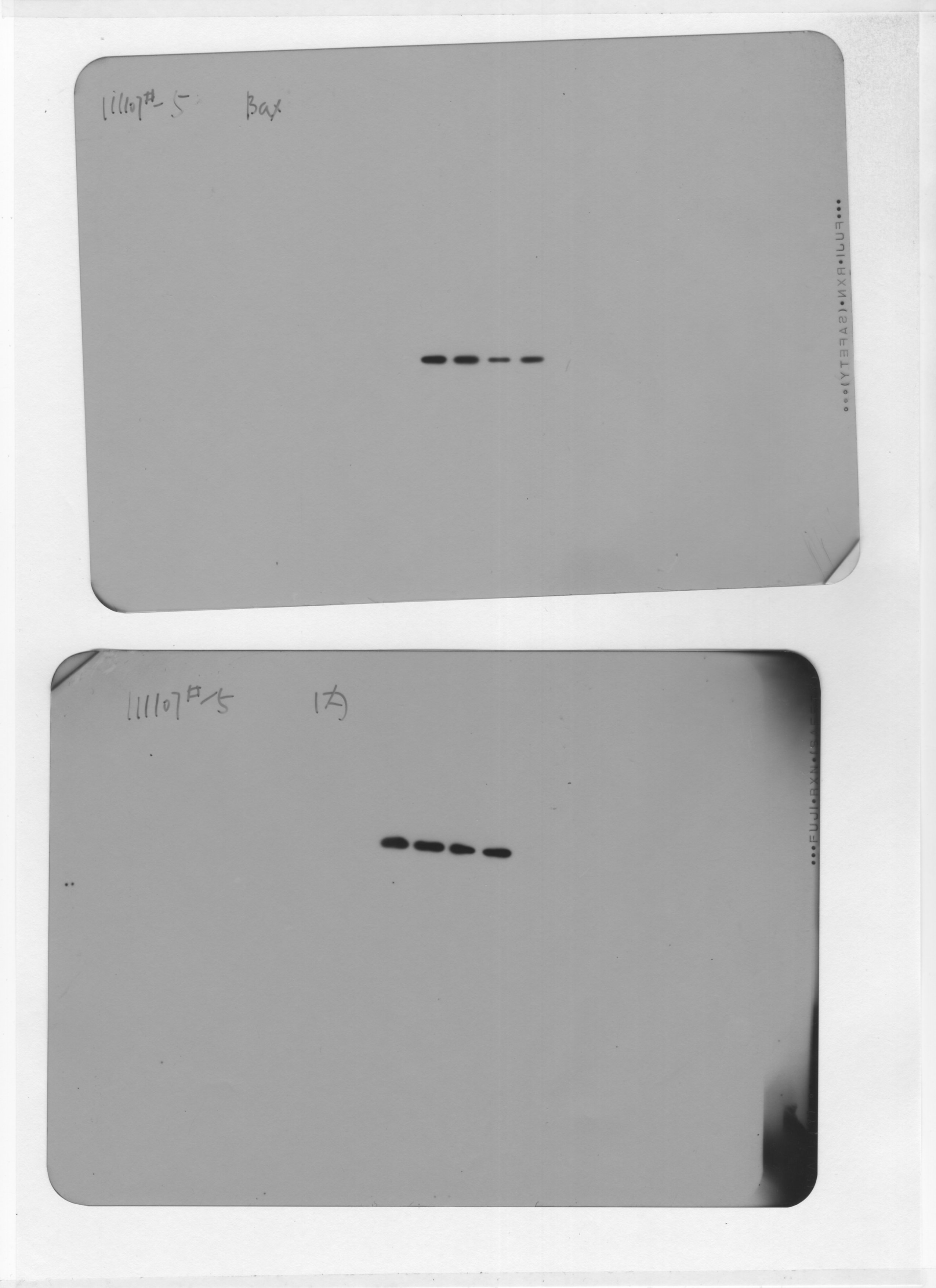


Caspase-3 /Cleaved Caspase-3

From left to right ：control；si-NC；OSR1-siRNA-3；OSR1-siRNA-3+BAY 11-7082；


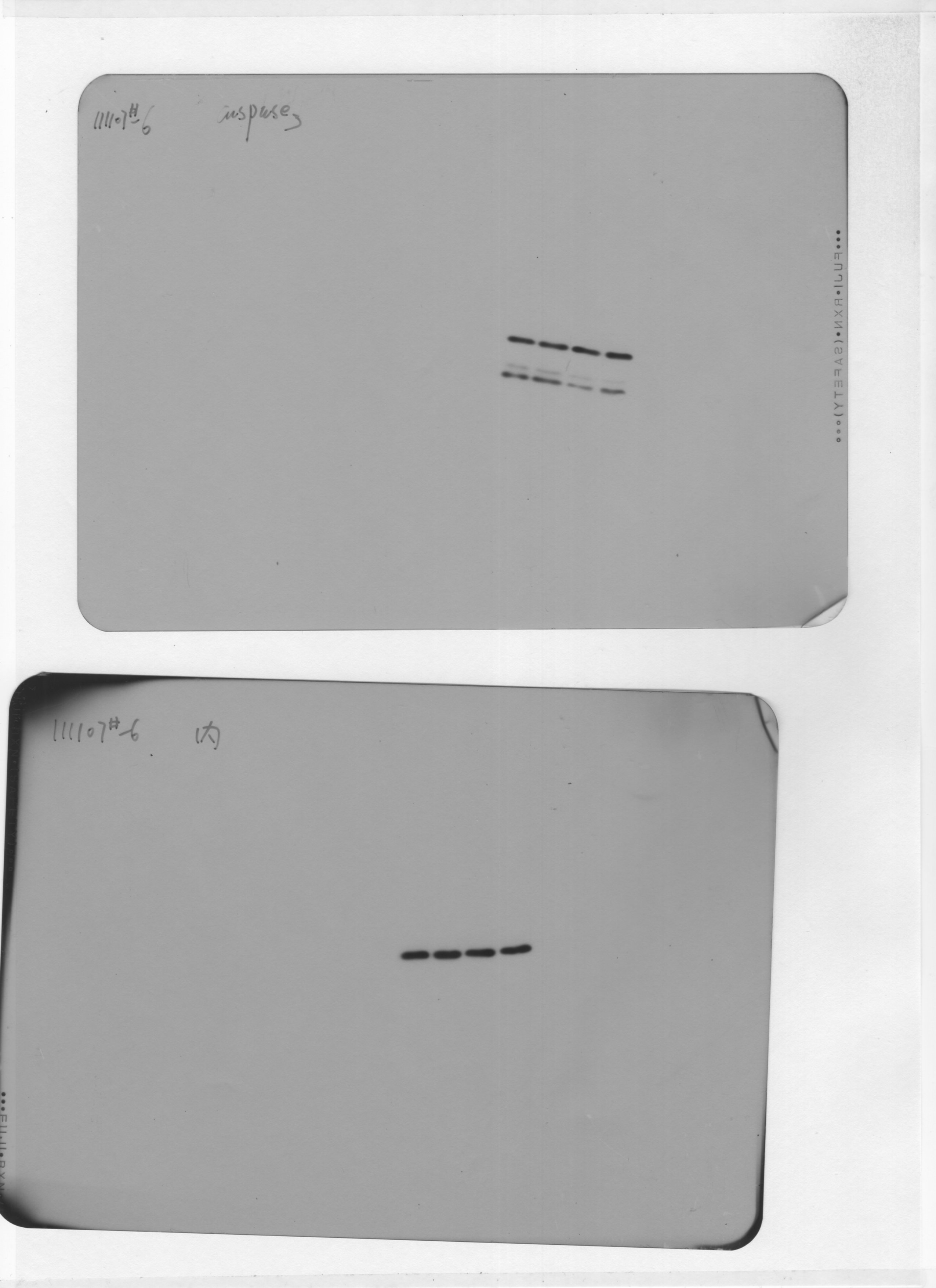


β-actin

From left to right ：control；si-NC；OSR1-siRNA-3；OSR1-siRNA-3+BAY 11-7082；


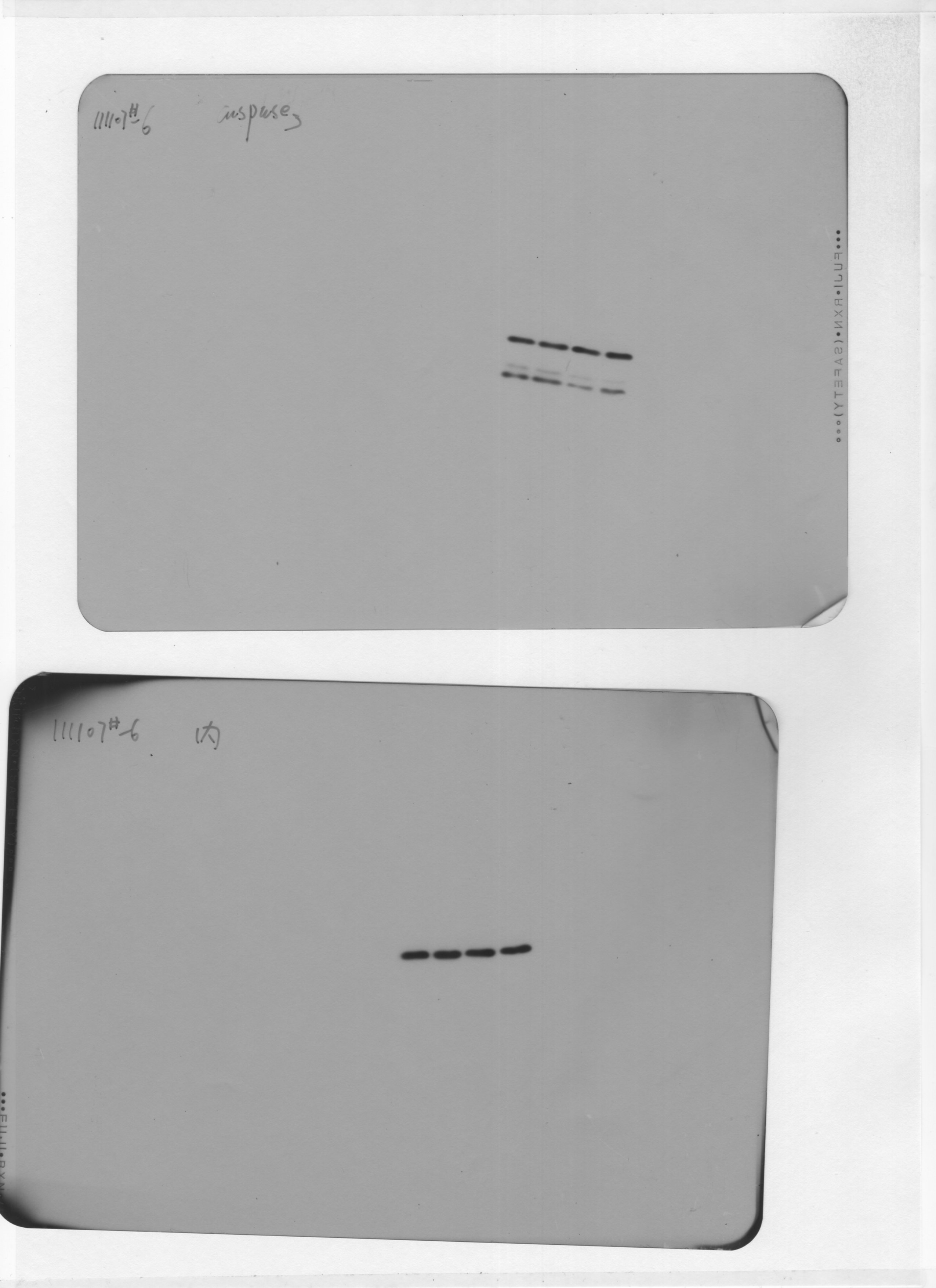

Supplement: Supplementary file 1 — Additional file1 (DOCX 27138 KB) [file 12672_2023_778_MOESM1_ESM.docx]
